# Unleashing the combined potential of bio-based carbon dioxide removal in Germany’s path to net zero from the bioenergy system perspective

**DOI:** 10.1016/j.isci.2026.116852

**Published:** 2026-08-06

**Authors:** Mohammad Sadr, Danial Esmaeili Aliabadi, Matthias Jordan, Ronja Wollnik, Daniela Thrän

**Affiliations:** 1 Helmholtz Centre for Environmental Research - UFZ, Permoserstraße 15, 04318 Leipzig, Germany; 2 Deutsches Biomasseforschungszentrum gGmbH–DBFZ, Department of Bioenergy Systems, 04347 Leipzig, Germany

**Keywords:** climate change mitigation, techno-economic modeling, carbon dioxide removal, BECCS, natural sinks

## Abstract

Carbon dioxide removal (CDR) is an essential measure for achieving national and international climate targets, complementing emission reductions. Germany aims for net-zero greenhouse gas emissions by 2045 and net-negative emissions thereafter. Nonetheless, current deployment levels are still a far cry from what is needed. This study employs a comprehensive modeling approach to examine the regional deployment of bio-based CDR in Germany, integrating techno-economic and regional conditions. We focus on two key CDR approaches: technical and natural sinks, thereby coupling energy and non-energy sectors and providing a cross-sectoral view. The selected CDR concepts are implemented in the extended bioenergy optimization model (BENOPTex) to assess their deployment for achieving climate targets cost-effectively in Germany. Our analysis demonstrates that a co-deployment strategy of technical and natural sinks, as opposed to deploying solely technical sinks, can enhance overall CDR potential by at least 50% while cutting removal costs, achieving around ∼110 Mt CO_2_ removal annually by 2050.

## Highlights

• Analyzing the cost-effective potential contributions of over 20 bio-based CDR options
• BECCS and natural sinks can together remove ∼110 Mt CO_2_ annually in Germany by 2050
• A residual gap of more than 80 Mt CO2 persists for Germany’s emissions target in 2045
• Combined removal strategies boost Germany’s CDR potential by 50% and reduce its costs

## Graphical abstract

**Figure:**
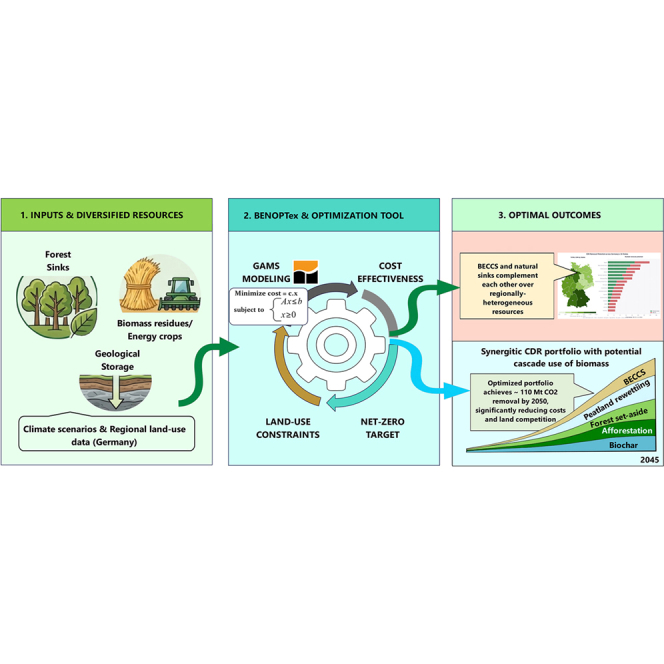

## Teaser

Environmental science; global carbon cycle; energy systems

## Introduction

Over the last decade, the global temperature has increased at an unprecedented rate, mainly due to high greenhouse gas (GHG) emissions,^1^ with CO_2_ being the most significant GHG driving global warming. The Intergovernmental Panel on Climate Change (IPCC) emphasizes that limiting global warming to 1.5°C–2°C will require not only deep emissions reductions but also the deployment of carbon dioxide removal (CDR) technologies at a multi-gigaton scale. CDR is now emerging as a front-and-center element, complementing mitigation efforts to achieve climate stabilization by mid-century.^2^^,^^3^

CDR refers to technologies, practices, and approaches that remove atmospheric CO_2_ and store it durably in vegetation, soils, geological formations, the ocean, or manufactured products.^4^ Numerous CDR methods have been assessed and proposed for large-scale deployment due to their promising potential. They include, but are not limited to, afforestation/reforestation (AR), enhanced weathering (EW), soil carbon sequestration, biochar, direct air carbon capture and storage (DACCS), bioenergy with carbon capture and storage (BECCS) (BECCS captures biogenic carbon and store it underground in a geologic formation while converting biomass into renewable heat, power, and biofuels^5^), ocean fertilization, and wetlands restoration.^6^^,^^7^ CDR concepts can be categorized as technical sinks (e.g., BECCS, DACCS, long-lived biomass materials) and natural sinks (e.g., afforestation, reforestation, soil carbon sequestration), exhibiting significant variations in key aspects, inter alia, the underlying removal process, associated costs, co-benefits, timescales for carbon storage, technological maturity levels, potential adverse side effects, governance requirements, and their ability to contribute to mitigating global warming. For instance, BECCS offers scalable, permanent storage and delivers co-benefits through the production of energy (e.g., electricity, heat) and removing CO_2_, but comes with trade-offs, including high infrastructure investment and limitations imposed by the sustainable availability of biomass feedstock and land. In contrast, natural sinks (e.g., forest management, peatland rewetting) provide ecosystem co-benefits such as biodiversity and water regulation but are also fundamentally constrained by land availability and can be vulnerable to reversals.^5^

Current CDR efforts (∼2 Gt CO_2_/yr) rely heavily on conventional methods (“Conventional CDR” refers to CDR on land that is nature-based. This category includes methods that enhance natural carbon sinks and are already practiced at scale (e.g., AR, soil carbon sequestration in croplands and grasslands, peatlands and wetlands restoration). Activities that use technology to create a carbon sink, such as BECCS and DACCS, are referred to as “novel CDR” activities^8^), however, novel approaches (The current contribution of novel CDR approaches remains relatively small of ∼0.0013 Gt per year^6^) must scale rapidly to meet climate targets. It is almost universally accepted in global scenarios that both technical and natural sinks are required under ambitious climate targets.^6^^,^^9^^,^^10^ Yet, their optimal co-deployment and trade-offs within a spatially explicit national energy system model remain less explored, particularly in countries with dense land-use conflicts such as Germany. To close this gap, Migo-Sumagang et al.^11^ suggest that integrated assessment models (IAMs) must be complemented by additional computing tools that can prescribe optimal decisions rather than merely describe them through lowering resource consumption, carbon footprint, and waste, in the implementation of CDRs. A lack of studies providing computational decision support remains in deploying optimal CDR portfolio.^12^^,^^13^ Cheng et al.^14^ investigate two scenarios, under which bioenergy expansion and AR compete to limit global warming. While the AR-focused scenario is better at removing CO_2_ with more certainty, it may lead to a relatively warmer regional climate than bioenergy expansion; therefore, the authors suggest that regional characteristics should be considered for effective climate mitigation when choosing AR or bioenergy.

Although IAMs can cover a broader geographical scope, thereby showing the telecoupling effects among different regions and sectors in the world,^15^^,^^16^^,^^17^ they lack spatial resolution^11^^,^^18^ to assess regional trade-offs in countries such as Germany, where land competition and policy frameworks shape feasibility.^19^ Compared to these global-scale models, the proposed high-resolution, regionally explicit optimization model in this study can provide results that account for sub-national heterogeneity in biomass and land availability. Moreover, an integrated modeling of many CDR options will be needed to generate a consistent view when analyzing synergies and trade-offs. For instance, Deng et al.^20^ demonstrate that combining BECCS and biochar can cost-effectively increase CDR by 15%–400%, particularly in regions with poor CO_2_ transport infrastructure. Rickels et al.^21^ also found that most IAMs typically focus on BECCS and AR applied individually rather than in combination. By combining BECCS, DACCS, AR, and EW, Strefler et al.^22^ stipulate that a balanced portfolio of CDR is best from a regional perspective. It would be more feasible and sustainable to deploy a portfolio of options at substantial, but smaller scales, rather than a single large option.^21^ Migo-Sumagang et al.^13^ present a multi-period optimization model that maximizes the net present value of a CDR portfolio based on carbon prices, CDR costs, and discount rates. It provides a framework for evaluating the economic viability of various CDR combinations over time, considering multiple environmental footprints, technological readiness, and ecological discount rates.

**Figure 1:**
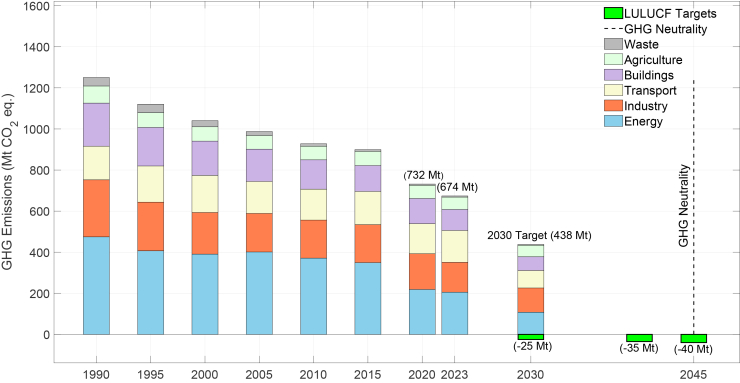
Germany’s sectoral GHG emissions and the targets through years

National policy frameworks play a pivotal role in shaping the scale, scope, and timing of CDR deployment, particularly in aligning technological pathways with legal targets and institutional priorities. Effective CDR deployment hinges not only on technological feasibility but also on policy frameworks that incentivize coordination across sectors and regions. As part of the Paris Agreement, Germany has set a goal of achieving net-zero emissions by 2045, five years ahead of the EU target. Moreover, it seeks to establish intermediate targets for technical carbon sinks and a net-negative GHG emissions target in 2060 to reverse climate change. In 2019, Germany passed its first national climate law, which was amended in 2021 and 2024, describing the annual emission budget for individual sectors such as energy, industry, and transportation, as illustrated in Figure 1.^23^^,^^24^ While Germany’s pathway to achieving net-zero CO_2_ emissions predominantly relies on extensive emission avoidance and reduction strategies, the remaining residual emissions necessitate the deployment of CDRs, which play a critical role in balancing any unavoidable residual emissions.^25^ The current climate law stipulates that Germany will strive to achieve negative emissions by 2050, but it falls short of specifying any targets. The Climate Action Law in Germany only specifies net-removal targets for land use, land use change, and forestry (LULUCF), which call for 25, 35, and 40 Mt CO_2_ removal by 2030, 2040, and 2045, respectively. Among natural sink options, peatland rewetting, for example, offers significant negative emission potential in Germany. Drained peatlands currently account for approximately 7% of national emissions. Rewetting initiatives could provide substantial CDR benefits while supporting biodiversity goals. Given its large mitigation potential and political attention in Germany, peatland restoration is a key element of the CDR portfolio analyzed.^26^ A Carbon Management Strategy is also under development in Germany to address emissions at the source before entering the atmosphere.^27^^,^^28^ These national goals frame the necessity for integrating CDR deployment into climate action; however, the law lacks explicit CDR deployment pathways, creating uncertainty for bio-based strategies.

Despite BECCS technologies being the salient CDR options for reaching climate targets, their combined deployment remains poorly understood—especially in industrialized nations with dense land-use conflicts such as Germany.^28^ This is critical because over-reliance on single CDR approaches risks policy lock-in (e.g., excessive BECCS dependency straining biomass supplies), and regional disparities in land availability and infrastructure could exacerbate inequities in CDR implementation. Hence, the following critical gaps persist in the literature:

1. Existing studies rarely model these synergies at spatially explicit scales, and
2. Germany’s current policies lack integrated frameworks to optimize such hybrid portfolios.

This disconnect between technical potential and policy implementation underscores the need for a systematic assessment of co-deployment strategies, addressing not just removal potentials but also regional bottlenecks. To this end, using a techno-economic evaluation in the extended bioenergy optimization model (BENOPTex),^29^^,^^30^ we examine the effectiveness of the most prominent bio-based CDR options—considering removal potential, deployment cost, removal intensity, and cost of removal—in Germany’s path to its net-zero target.

BENOPTex is a technology-rich cost-optimization model that integrates techno-economic and political factors of Germany’s energy and non-energy sectors at the federal state level, respecting regional biophysical constraints (land, biomass). However, similar to many partial equilibrium models,^31^ it does not endogenously capture market feedback (e.g., wood demand elasticity, rebound effect), social acceptance, carbon leakage, permanence concerns for temporary storage, or other real-world challenges. The outcomes of such detailed models can serve as a guiding light for decision-makers in adjusting their strategies and aligning actions with long-term goals.

By combining BECCS and natural sinks (energy and non-energy sectors), we expand our CDR portfolio and shift away from the current focus on annual sectoral emission reduction targets to a cross-sectoral view, lowering the system’s removal cost, which is a hurdle for the expansion of CDR. We particularly focus on terrestrial CDRs, including BECCS, forest management, agricultural practices, peatlands rewetting, and paludiculture cultivation, taking into account regional characteristics, as their performances is affected heavily by a complex interplay of bio-geophysical, techno-economic, and socio-political elements. In this study, regional variations in biomass/land availability, land use, socio-economic factors, policy, and regulatory environment make a regional approach essential for optimizing CDR deployment.^32^^,^^33^^,^^34^ Regional variations in CDR deployment potential arise from differences in biomass yields (e.g., miscanthus, sugar beet), land availability for afforestation, peatlands rewetting or soil carbon sequestration, existing land-use patterns (agriculture vs. forest), the price of energy crops based on the gross regional product (GPR), and expanding rate based on their relative amount in each region (i.e., federal states of Germany). Therefore, increasing the model’s spatial resolution through region-specific factors is another objective.

The economic viability of CDR strategies is highly sensitive to carbon pricing regimes. In Germany, carbon prices (€55/tCO_2_ in 2025 under the national fuel emissions trading system (In order to supplement the EU Emissions Trading System (EU ETS), the German National Emissions Trading System (nEHS) was created as a carbon pricing mechanism. It concentrates on industries that are not included in the EU ETS, mainly transportation and heating^35^)), and is projected to rise from 2026 onwards based on the auction mechanism,^36^ with further increases expected to meet net-zero targets.^37^ This creates critical incentives for CDR deployment but also introduces challenges. Higher prices may accelerate fossil fuel phase-out^38^ but may disproportionately burden sectors reliant on nature-based solutions due to land competition.^19^ Finally, by conducting a sensitivity analysis on uncertain parameters such as carbon pricing and carbon credit prices at the national system level, we identify the main cost drivers and deployment barriers affecting the optimal mix of CDR technologies across regions and sectors.

## Results

This section presents the outcomes of our analyses, structured around the central research questions of the study:

1. What is the cost-effective removal potential of natural sink-based CDRs in Germany, and how do they compare to policy targets?
2. How can the co-deployment of bio-based technological (e.g., BECCS) and natural sink CDR options be optimized to meet national climate targets in a cost-effective manner?
3. What are the major financial factors and constraints affecting the deployment of different CDR pathways?

To answer these questions, we first evaluate the removal potential and deployment trajectory of key natural sinks under cost and land availability constraints. We then analyze a nationally cost-optimal deployment pathway that integrates BECCS and natural sinks across energy and land-use sectors, comparing it to national emissions targets to identify residual gaps. Finally, we assess the system-wide costs and conduct a sensitivity analysis to highlight the most influential economic and policy parameters shaping future CDR deployment.

### Natural sinks and LULUCF targets

**Figure 2:**
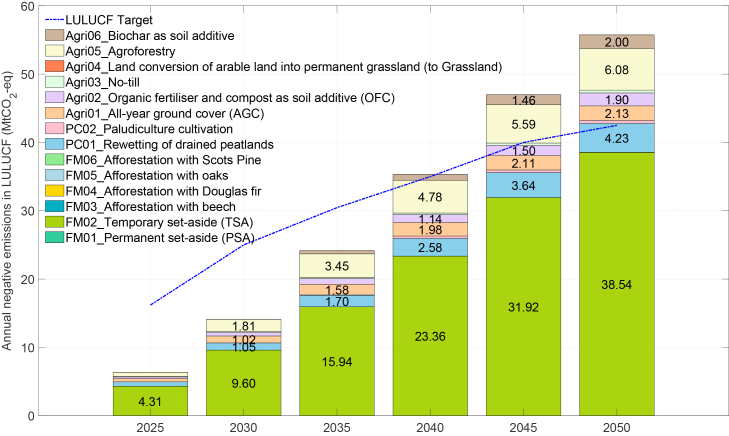
Cost-optimal deployment of natural-sink CDR options contributing in the German LULUCF sector Assumes a carbon price rising from €20/tCO_2_ in €2020 to €250/tCO_2_ by 2050, based on the central scenario used in the optimization model. The red thin bars between the dashed trend lines and the emission reduction mix indicate the amount that needs to be purchased, while the green bars represent the reduction achieved beyond the determined targets.

Figure 2 depicts the removal potential of various natural sinks over time compared to Germany’s LULUCF target. According to results, natural sinks have the potential to meet German LULUCF targets by mid-century (i.e., 2040–2050), but may fall short in meeting these targets in the near term. By mid-century, they could remove almost 55 Mt CO_2_, given a modest growth of carbon price to around €250 per tCO_2_ in 2050. The model initially struggles to meet the LULUCF targets before 2040 due to the limited growth rate of natural sink deployment (e.g., afforestation at less than 1, 000 ha/year or peatland rewetting at more than 2, 000 ha/year). However, the targets are met and exceeded in later years, with removals surpassing the target by approximately 6 Mt CO_2_ in 2045 and 13 Mt CO_2_ in 2050. The model exceeds the LULUCF targets in later years because it optimizes for the entire energy-land-use system. As the national GHG budget tightens and carbon prices rise, the negative emissions provided by cost-effective natural sinks (e.g., temporary set-aside (TSA)) become increasingly valuable. Deploying them beyond the minimum LULUCF requirement provides a cost-optimal strategy to offset hard-to-abate emissions in other sectors and reduce reliance on international carbon credits, thereby helping to meet the overall national climate targets more efficiently.

The most significant contributor among natural sinks in our analysis is the TSA of beech forests, resulting in the removal of 38.5 MtCO_2_ in 2050. It outcompetes the *permanent set-aside* (PSA), as an alternative option for the same beech forest area. This equates to setting aside the entire 2.39 Mha of beech forest area gradually. This CDR approach involves halting commercial forestry operations in designated forest areas for a prolonged period, allowing trees to grow beyond their typical economic rotation age and thus accumulate significantly more carbon in biomass and soils. Under the model’s cost-optimization logic, the TSA of all German beech forests emerges as the most cost-effective natural sink option, representing an upper technical-economic bound, as it assumes no considerations beyond biophysical availability and cost. In reality, this scale of set-aside would face challenges, including impacts on the domestic wood supply chain and the risk of carbon leakage, which are not endogenously captured in the BENOPTex model. Hence, this value should be interpreted as a technical upper bound under idealized conditions rather than a forecast; however, we conducted a sensitivity analysis over the share of beech forests dedicated to set-aside concepts in Figure S6. Our analysis limits the set-aside potential to beech forests specifically due to their distinct ecological and economic characteristics. Beech trees are native to Germany and have a high capacity for long-term carbon sequestration. Furthermore, beech forests represent a substantial and well-mapped portion of Germany’s forest estate,^39^^,^^40^ allowing for robust regional potential assessment. This focus on beech forests is a modeling choice based on current data availability and lower implementation risk, but it implies that the overall potential for forest-based CDR could be higher if other tree species were also included in future assessments. In our model, temporary (i.e., TSA) and PSA options for beech forests are technically differentiated only by their investment cost, which reflects the higher opportunity cost of permanently abandoning timber revenue. Since both provide identical annual CO_2_ removal within the model’s horizon (to 2050), the cost-optimal solution consistently selects the cheaper TSA option. The PSA pathway is included to ensure a complete representation of policy options and to facilitate future analysis exploring scenarios with extended time horizons, different opportunity costs, removal rate, or policies explicitly mandating permanent protection. However, it is crucial to note that, in reality, TSA is expected to have a slightly lower long-term climate benefit due to harvesting wood and consumption in different sectors (For instance, releasing CO_2_ back to the atmosphere through combustion in the energy sector or decomposition); therefore, its cost-effectiveness should be reevaluated in a framework that extends beyond 2050, possibly until the end of the current century.^41^ Nevertheless, obtaining the required data through to the end of the century poses significant challenges for such detailed optimization models.^42^

On the other hand, afforestation options might not provide the biggest contribution due to higher cost and limited land availability. Unlike BECCS, natural carbon sinks are inherently susceptible to carbon release, and they are also vulnerable to disturbances (e.g., wildfires, droughts, pests). Moreover, the potential for carbon leakage, where not harvesting in set-asides is merely displaced to other forest areas, may negate any net carbon benefit. Hence, conducting risk assessment might be a valuable direction for future research. Finally, we assume the current beech forest will be gradually associated with the TSA or the PSA options. Nonetheless, the government can opt for a more radical decision of declaring all forests as set-asides, thereby reaching the near-term LULUCF targets.

Model results also demonstrate significant potential of peatland rewetting for CO_2_ removal, contributing to national GHG reduction targets. While the estimated CO_2_ removal in 2030 (1.05 Mt CO_2_, requiring 92,000 ha rewetting) appears modest compared to the too ambitious 5 Mt target outlined in the national peatland conservation strategy, the mid-century removal potential is substantial (4.23 Mt CO_2_), achievable through rewetting approximately another 684,000 ha of drained peatlands. The model is based on an initial assumption of 80,000 ha of rewetted wetlands in 2020 (see Equations 15, 16, and 17). The outcomes indicate that paludiculture cultivation on organic soils, when integrated with BECCS technologies, could also offer significant potential for removal. Paludiculture can serve as a valuable feedstock for producing heat and electricity within BECCS technologies. While the initial impact by 2035 is projected to be limited at 0.1 Mt CO_2_eq, the long-term benefits are significant, reaching 0.53 Mt CO_2_eq by 2050. This increase is primarily driven by the expanding role of BECCS technologies in the middle of the next decade, coupled with the increasing penetration of other renewable energy sources, gradually displacing conventional plants such as coal-fired ones.

By 2050, agricultural practices have the potential to remove approximately 12.5 Mt of CO_2_. Among these practices, *“agroforestry”* stands out with the highest potential, estimated at around 6 Mt by mid-century. The concept of *“all-year ground cover”* (AGC) follows with a potential removal of 2.1 Mt CO_2_, and *“organic fertilizers and compost”* contribute about 1.9 Mt of CO_2_. In contrast, *“no-till”* and *“conversion of agricultural land to grassland”* do not exhibit significant removal potential in these results. This is primarily due to the low growth rate associated with no-till farming, the high cost, and the slow pace of converting agricultural land to grassland. The *“biochar application,”* which is estimated to remove 2 Mt of CO_2_, is currently limited by the model assumption that biochar production must meet the demand within only the agricultural sector (see Equations 20, 21, and 22). Additional optimization results are presented in the Supplementary Material (Tables S1 and S2). Lastly, one can conclude that since natural sinks are unable to meet near-term targets in the LULUCF sector, it is unlikely that they will contribute substantially to additional removal capacity in other sectors if those sectors fail during this period.

### The combined BECCS and natural sinks

**Figure 3:**
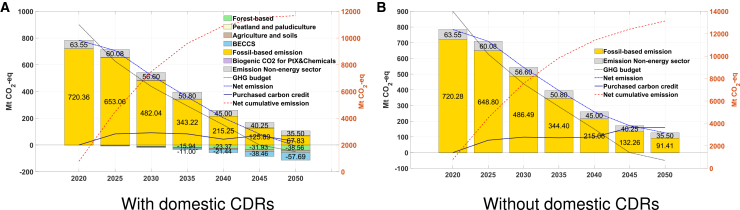
Cost-optimal deployment of BECCS and natural sink CDR options across German regions by 2050 Cost-optimal deployment of BECCS and natural sink CDR options across German regions by 2050 with (A) and without (B) domestic CDR options. Results shown assume a national net-zero target by 2045, a carbon price rising from €20/tCO_2_ in €2020 to €250/tCO_2_ by 2050, and region-specific land and biomass constraints as defined in the optimization model. To prioritize the use of domestic resources for meeting climate targets, the price of carbon credits from outside sources was assumed to be 10% higher than the domestic carbon price for the same year.

Our finding underscores the need for a two-pronged approach to climate change mitigation: A rapid and substantial phase-out of GHG emissions across all sectors, coupled with a progressive and sustained increase in the deployment of CDR concepts. Figure 3A illustrates the contributions of combined BECCS and natural-sink CDRs toward achieving net-zero emissions in Germany by 2045 and net-negative afterward. While significant emission reductions are achieved through the transition to renewable energy sources and energy efficiency improvements, leading to less dependence on fossil-based sources, the results demonstrate a substantial reliance on CDR concepts, which contribute to the removal of ∼110 Mt of CO_2_ by 2050. Furthermore, the CDR portfolio also supplies ∼3 Mt of biogenic CO_2_ annually as a circular carbon feedstock. This CO_2_ is routed to power-to-X (PtX) pathways and the chemical industry, creating renewable fuels (e.g., biodiesel) and chemicals (e.g., methanol). This cascade use of carbon maximizes the value of biomass, supporting sectoral decarbonization while geological storage provides the net-negative balance required for long-term climate targets.

Nonetheless, the residual gap of approximately 43 Mt CO_2_ between the system’s modeled achievement (the blue solid line) and the target (the black dotted line) in 2050 highlights the cost-optimal role of CDRs in complementing deep decarbonization. While further fossil emission reductions—particularly beyond the 68 Mt remaining in 2050—are technically feasible, the model favors deploying CDRs (through BECCS ∼ 58 Mt CO_2_, natural sinks ∼55 Mt CO_2_, and 27 Mt CO_2_ by carbon credits/external CDRs) to offset hard-to-abate emissions and reach the targets when it is more economically efficient than pursuing a complete fossil phase-out. It is while Germany is projected to fall short of its net-zero target by 2045, with net emissions estimated at 83 Mt CO_2_, even after implementing 85 Mt of CDRs within the country that year. Closing this gap requires a more aggressive reduction of gross residual emissions and access to a larger CDR portfolio than the domestic bio-based options analyzed here.

The model results reveal a persistent gap between Germany’s GHG budget (target) and the projection of net emissions, both in the short and long terms. This outcome reflects the model’s allowance for purchasing international carbon credits as a cost-effective compliance option, which permits emissions to exceed the domestic GHG budget under certain economic conditions. While this flexibility results in a remaining cumulative emissions balance of approximately 11.68 Gt CO_2_ by 2050 (as shown by the secondary vertical axis), a stricter policy design-such as excluding external credits—could close this gap through deeper domestic mitigation or enhanced CDR deployment.

The concern that CDR deployment might be used to offset emissions rather than eliminate them at the source in a cost-optimization model with a single carbon price—potentially diminishing near-term decarbonization efforts—motivated a model run without domestic CDR technologies. This “mitigation deterrent” effect is a known risk in policy design.^43^ As illustrated in Figure 3B, the results reveal nearly 24 Mt more fossil-based emissions in 2050 without CDRs, while achieving net neutrality becomes more challenging with approximately 127 Mt CO_2_ residual emissions by 2050, compared to ∼103 Mt CO_2_ with CDRs.

Without CDRs, Germany risks substantial failure in meeting its 2045 net neutrality target, and net-negative emissions become infeasible beyond that. This highlights the critical role of CDR in counterbalancing residual emissions and indicates that they may not impede fossil emission reduction under this assumption. The seemingly counterintuitive result of higher residual fossil emissions in the absence of CDR arises from the model’s cost-optimization logic. When CDR technologies such as BECCS are available, they serve a dual purpose: They generate low-carbon energy (displacing fossil fuels in the energy system) while also removing CO_2_. Removing the CDR options eliminates these efficient pathways. Consequently, the model must meet the emission budgets through more expensive means, thereby increasing the total system cost. Without CDR, the cost-optimal solution relies more heavily on purchasing international carbon credits, which allows for a higher level of residual domestic fossil emissions to persist. This result highlights that CDR technologies are not merely a compensatory measure but can be integral to direct decarbonization of the energy system. However, an increase in the price of carbon credits/external CDRs could alter the energy system’s cost-optimal pathway, as the lack of external CDRs would necessitate a greater focus on reducing residual emissions and increasing domestic CDRs. Conversely, the availability of low-cost external CDRs could potentially weaken domestic efforts to curb fossil fuel emissions. The section on the impact of carbon credit goes into greater detail.

**Figure 4:**
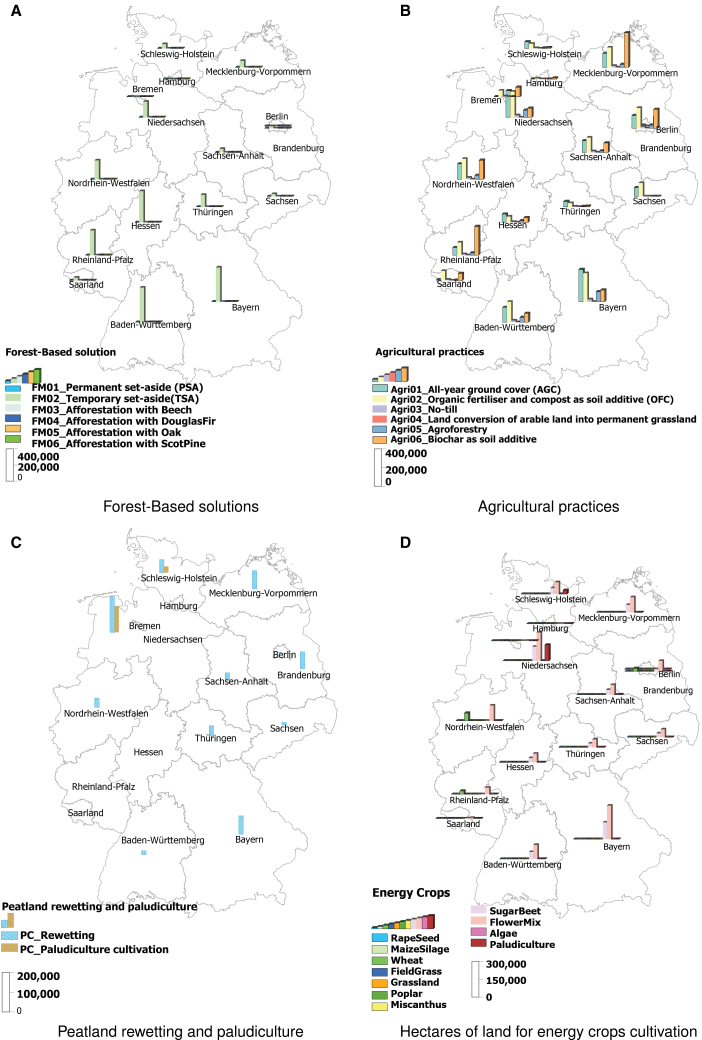
The regional hectares of land used at the end of the planning horizon (2050)

The co-deployment of BECCS and natural carbon sinks pathways achieves synergistic system performance through strategic resource allocation and complementary operational characteristics. This strategy leverages three key mechanisms: (1) efficient resource utilization, where BECCS and natural sinks complement each other, resulting in less constrained resource use; (2) explicit management of resource trade-offs (via Equation 19), which optimizes the balance between forest set-aside for sequestration versus residue extraction for carbon-negative energy generation; and (3) spatial optimization that matches regionally heterogeneous resource endowments to CDR-type comparative advantages (see Figure 4), accessing removal potential across diverse land types unavailable to single-technology approaches.

In terms of total system cost, the results indicate that incorporating CDR reduces the cost of achieving the climate neutrality target. Natural sinks can enhance the cost-effectiveness of CO_2_ removal in the systems by providing more options. The integration of natural sinks with BECCS can substantially lower the system cost of removal, reducing it from an average of €200/t CO_2_ with BECCS alone to €150/t CO_2_ (see Equations 25 and 26). Finally, in terms of removal intensity, BECCS concepts outperform natural-based concepts, with an average of 26 t CO_2_ per hectare versus 8 tons per hectare in 2050 (This includes an average of 13.8 t CO_2_ per ha for the forest option and 1.8 t CO_2_ per ha for the agricultural practices)(see Equations 27 and 28).

### Regional distribution of CDRs

Adopting a regional perspective is essential for forming a realistic cost-optimal assessment of Germany’s overall CDR capacity. Examining the results at the state level in Figure 4 reveals the distribution of various CDR concepts in relation to the required land area. While some states, such as Baden-Württemberg, are well-suited for forest-based solutions, they may be less suitable for rewetting and paludiculture production. Forest-based solutions are particularly effective in regions with large existing forest areas, as they leverage existing ecosystems and require minimal land conversion. However, the availability of suitable land for afforestation and forest management may be limited in densely populated or agriculturally intensive regions (see Figure 4A). Agricultural CDR practices, such as *“agroforestry,” “all-year ground cover,*” and *“organic fertilizer”* application, are more evenly distributed across states with significant agricultural activity. For example, states such as Niedersachsen and Nordrhein-Westfalen, which have large agricultural sectors, show higher potential for these practices. Nevertheless, the implementation of agricultural CDR practices is constrained by land-use competition with food production and other economic activities. This highlights the need for integrated land-use planning to balance CDR goals with agricultural activities (see Figure 4B).

As shown in Figure 4C, *“Peatland rewetting”* and *“paludiculture”* are highly region-specific, with the greatest potential in states such as Mecklenburg-Vorpommern, Niedersachsen, and Brandenburg, where large areas of drained peatlands are available for rewetting. These regions are less suitable for forest-based solutions due to the limited availability of beech forest areas. However, rewetting peatlands offers significant co-benefits in these regions, such as biodiversity conservation, making it a valuable component of Germany’s CDR strategy.

Figure 4D illustrates the regional distribution and allocation of land for the cultivation of energy crops, as optimized in our model to support BECCS. Sugar beet, paludiculture, miscanthus, and flower-mix are cornerstone feedstocks among others, with regionally tailored deployment. Sugar beet is widely cultivated across regions such as Niedersachsen, Bayern, and Nordrhein-Westfalen, reflecting its high biomass yield and compatibility with existing agricultural systems. Paludiculture cultivation on rewetted peatlands (e.g., Schleswig-Holstein and Niedersachsen) offers dual benefits of CO_2_ removal in restored peatland and providing feedstock for BECCS. The relative share of traditional energy crops (e.g., RapeSeed, MaizeSilage, and Wheat) has shifted in favor of the lignocellulosic biomass crops (e.g., Miscanthus and Flower-mix), which are more efficient and well-suited for BECCS integration (e.g., biomethane with CCS, combustion heat and electricity with CCS), serving as critical inputs.

### Sensitivity analysis

The deployment of CDR technologies, such as BECCS and natural sinks, is subject to significant uncertainties, including fluctuations in carbon pricing, availability of external CDRs/carbon credits, land availability, deployment pace, technological readiness, and policy support. Sensitivity analysis allows us to explore how these uncertainties influence the optimal deployment pathways, providing valuable insights for policymakers and stakeholders aiming to achieve Germany’s net-zero targets.^19^^,^^44^^,^^45^ To assess the sensitivity of our findings, we vary key parameters within plausible ranges rather than employing the scenario-based approach. The sensitivity analysis allows us to quantify the impact of uncertainties on the optimal CDR portfolio, providing a comprehensive understanding of the trade-offs and synergies between different CDR options.

### Carbon price impact on CDRs’ deployment

To assess the impact of carbon pricing on the deployment of CDR technologies, a sensitivity analysis was conducted by varying the carbon price factor (*α*). The *α*-factor represents a multiplier applied to the base carbon price, with values set at 0.5, 1, 2, 3, and 4, corresponding to 50%, 100%, 200%, 300%, and 400% of the reference carbon price at each year, respectively. The model framework incorporates an interplay between fossil emission reduction, renewable energy utilization (e.g., via PtX), CDR deployment, and the availability of external carbon credits. In the baseline scenario (*α* = 1), the cost of carbon credits is deliberately set at 110% of the domestic carbon price to incentivize the utilization of domestic mitigation resources.

**Figure 5:**
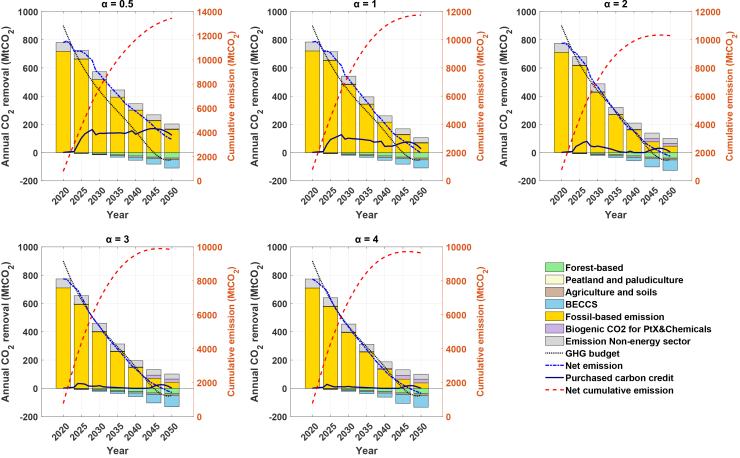
Carbon price system-wide impacts on emissions and CDR deployment The *α* factor is a multiplier that adjusts the base carbon price (*α* = 1), with values set at 50%, 100%, 200%, 300%, and 400%, respectively. The model assumes a carbon price rising from €20/tCO_2_ in €2020 to €250/tCO_2_ by 2050, following the central scenario used in the optimization model.

Figure 5 presents a sensitivity analysis examining how varying carbon prices influence the entire energy system from the bioenergy system point of view. Fossil-based emissions decline steadily as carbon prices rise, but residual emissions persist (38–163 Mt CO_2_/year by 2050), even at high prices. This reflects hard-to-abate sectors (e.g., transport and industry) where alternatives remain costly. This further highlights that the carbon price pathway implemented in this model alone may not fully phase out fossils without complementary solutions (even though we let coal be phased out by 2038 and hard coal by 2030). Our findings indicate that BECCS scales with carbon prices, ranging from 57 to 80 Mt CO_2_/year by 2050 from low to high prices. In contrast, natural sinks provide a near-constant removal potential (55–57 Mt CO_2_/year) across carbon prices (see Figure S5), reflecting both their cost-effectiveness and biophysical limits such as finite suitable land (e.g., only 2.39 Mha beech forests available for set-aside or 1% of grassland for afforestation), slow sequestration and growth rates (see Table S1).

The net emissions in Figure 5 (the dashed blue line) decrease significantly as carbon pricing increases, indicating a shift toward more aggressive decarbonization strategies. It underlines that Germany turns net-negative by 2050 under high carbon prices, when domestic CDRs such as BECCS and natural sinks collectively offset residual emissions. Higher carbon prices are also correlated with a decreased demand for external carbon credits or a reduced reliance on CDRs outside Germany. This outcome is the artifact of the modeling logic. In the framework, the price of international credits is set at a premium (110%) to the domestic carbon price. Hence, as the carbon price rises, the cost of importing credits increases faster than the cost of deploying domestic CDR options. The model, seeking the least-cost pathway, responds by maximizing the use of cheaper domestic resources before resorting to more expensive international credits. However, at low prices, net emissions remain positive (93 Mt CO_2_/year in 2050 for *α* = 0.5), failing climate targets via domestic potentials. The cumulative emissions (red line) are substantially reduced over time under high carbon prices. Under the baseline trajectory (*α* = 1), the cumulative emissions peak around 2045–2050 before declining. By 2050, cumulative emissions reach approximately 11.8 Gt CO_2_. Higher carbon prices (i.e., 2 ≤ *α* ≤ 4) flatten the curve more aggressively. The cumulative emissions are reduced by almost 18% compared to the baseline by 2050. The plateau occurs 5–10 years earlier with stronger carbon pricing before it begins to decline.

The analysis underscores the effectiveness of carbon pricing in reducing emissions and promoting CDRs. One can see that fossil fuel emissions decline sharply with increasing carbon price, whereas CDR deployment responds more moderately. In high-price scenarios, the majority of net emission reductions stem from fossil fuel abatement, with a transition from the baseline to the highest carbon price yielding a 22% increase in CDR deployment compared to a 43% reduction in fossil emissions. However, some fossil fuel use might still remain in the system, which is likely to be offset by the deployment of BECCS and other CDR measures. On the other hand, high carbon prices impose high costs on the system. According to our model settings, Germany is unlikely to meet its net-zero target by 2045 under the baseline scenario (*α* = 1) without relying on carbon credits totaling 70 Mt of CO_2_. While international credits provide short-term flexibility, which allows them to serve as a substitute for domestic action within this modeling framework, their availability creates a risk of delayed investment in domestic capital-intensive options, including renewables (e.g., PtX and biomass plants) and CDRs. This occurs as the model’s cost-optimization mechanism leverages the cheaper short-term option (credits) and defers more expensive domestic investments. This “mitigation deterrence” effect is a recognized risk primarily due to the expectation or anticipation that future, uncertain CDR technologies will provide flexibility in achieving climate targets, thereby allowing delays or reductions in immediate emission mitigation efforts. Additionally, the broader political landscape, including deregulation agendas and policies aimed at simplifying climate policy, can inadvertently promote mitigation deterrence by making it politically easier to defer or dilute emission reduction efforts under the guise of future reliance on CDR capacities.^46^ At high prices (2 ≤ *α* ≤ 4), the gap closes, but the total system cost rises by 15–37%. Thus, the Cost-effectiveness threshold lies at *α* ≈ 1, where net emissions drop sharply without exponential cost growth.

**Figure 6:**
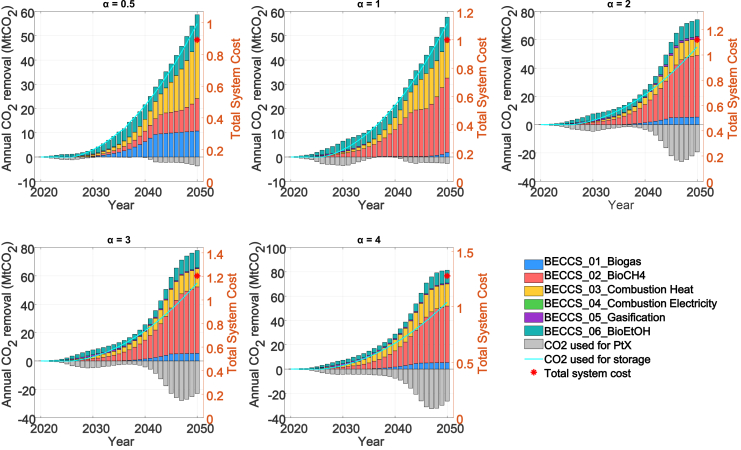
BECCS sensitivity analysis over carbon price The *α*-factor is a multiplier that adjusts the base carbon price (*α* = 1), with values set at 50%, 100%, 200%, 300%, and 400%, respectively. The right-hand side vertical axis shows the total system cost relative to the case where *α* = 1.

Focusing on technical CDRs, Figure 6 depicts the evolution of BECCS removal potential with respect to different carbon price scenarios. Our sensitivity analysis reveals that the carbon price trajectory is a paramount determinant of both the scale and composition of the BECCS portfolio. At lower carbon price levels (*α* = 0.5), the system achieves only modest levels of removal, peaking below 60 MtCO_2_/year, with deployment dominated by a limited number of more cost-competitive technologies such as bio-methane and combustion-based pathways. However, as *α* increases, a substantial expansion in BECCS capacity is observed, exceeding 80 MtCO_2_/year driven by the higher economic incentive for carbon removal. These scenarios foster a more diversified BECCS technology mix, incentivizing the deployment of a broader range of pathways, including gasification and bioethanol production with CCS. In particular, *“BECCS_05_Gasification”* exhibits notable growth in high-carbon-price scenarios, suggesting that these technologies are more cost-effective under stringent climate policies. Among the technologies, bio-methane with CCS (*“BECCS_02_BioCH4”*) emerges as a particularly resilient option, with its deployment increasing from 13 to 46 MtCO_2_/year as the carbon price rises. This results in a transition from fossil-based methane counterparts due to high penalties, with biomethane-based BECCS playing a significant role in district heating and industrial applications. In contrast to the stable profile of other pathways, *“BECCS_01_Biogas,”* and *“BECCS_05_Gasification”* exhibit high sensitivity to economic conditions, revealing their status as marginal or niche options.

Higher carbon prices accelerate the deployment of BECCS technologies, with significant investments occurring earlier (e.g., between 2030 and 2035) to maximize the economic benefits of carbon removal. In contrast, lower carbon prices delayed the deployment of BECCS, with most investments occurring closer to 2050, when the remaining carbon budget became more constrained, and the carbon price is higher at that time. It is also important to examine the total system cost and its trade-off with CDR deployment, as shown on the secondary vertical axis (at the right-hand side), where all values are standardized based on the carbon price used in this study (*α* = 1). The results suggest a monotonic, non-decreasing relationship between carbon pricing and total system cost. At lower carbon prices (*α* = 0.5), total system cost remains relatively low, but BECCS deployment is insufficient to support net-zero or negative emission targets, compounded by the high fossil emissions that remain in the system. The system relies more on fossil-based energy (see Figure 5), leading to higher emissions and lower removal potential. At higher carbon prices (*α* > 1), a substantial increase in BECCS deployment is observed compared to a low carbon price, leading to higher system costs due to the substantial penalties imposed on the fossil-based energy that persists in the system. When moving from *α* = 0.5 to 1, BECCS deployment remains almost stable, while total system costs increase by approximately 11%. At higher carbon prices, BECCS deployment increases slightly, but system costs increase sharply. Therefore, a moderate carbon price appears to be the optimal range, where CDR deployment is significantly enhanced without excessive increases in system cost (see Figure S3, Marginal Removal Cost Curve and system response to carbon price variation).

At higher carbon prices, the model also directs more captured biogenic CO_2_ from BECCS processes into PtX synthesis of fuels and chemicals, reducing reliance on fossil-based energy carriers. At lower carbon prices, the allocation to PtX remains limited, as the model prioritizes minimizing costs over maximizing CO_2_ utilization. Any captured CO_2_ not used for PtX is allocated to geological storage, which is classified as CDR and yields permanent negative emissions. This reveals a key trade-off: Biogenic CO_2_ is a valuable but limited resource that can either deliver permanent removals through storage or enable sectoral decarbonization via PtX. The balance between these options depends strongly on the carbon price signal and the availability and cost of storage.

Moreover, the impact of various carbon price multipliers on the deployment of natural sinks is analyzed in Figure S5. The results indicate that higher carbon prices incentivize increased CO_2_ removal through natural sinks. However, the overall response is limited, potentially due to both land availability constraints and the implementation rate of agricultural-based solutions despite their cost-effectiveness.

### The impact of carbon credit

**Figure 7:**
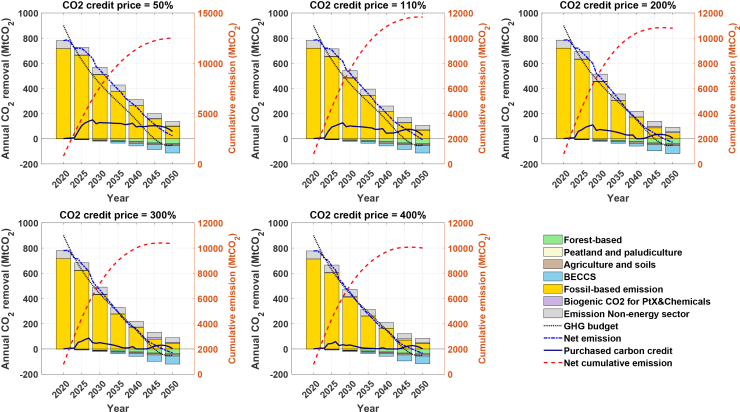
Carbon credit system-wide impacts on emissions and CDR deployment The CO_2_ credit price refers to the amount paid to purchase credits from outside the country to meet specified targets. To promote the use of domestic resources for climate targets, the price of external carbon credits is assumed to be 10% higher than the domestic carbon price in the same year in the base case.

This section explores how varying carbon credit prices (from 50% to 400% of the domestic carbon price) influence Germany’s emissions trajectory, CDR deployment, the total system cost, and reliance on external offsets. As illustrated in Figure 7, high carbon credit prices reduce reliance on imported offsets, shifting compliance to fossil emission reductions and domestic CDRs (BECCS + natural sinks). However, total CDR plateaus at ∼111 Mt CO_2_/yr (once the biogenic CO_2_ used in the model is subtracted) due to land/biomass constraints, forcing fossil emissions to bear the brunt of further reductions. Thus, while domestic CDR saturation limits its role, it remains critical to address this amount of non-abatable emissions and credit phase-outs. The domestic CDR deployment remains relatively stable between 113 and 117 Mt CO_2_/year by 2050, but fossil emissions experience a significant variation of 56 Mt CO_2_/year (98-56 Mt CO_2_/year) between scenarios with low and high carbon credit prices. Low/medium carbon credit prices (50%–110%) encourage reliance on external offsets and dominate CDR strategies, reducing the need for domestic actions and resulting in cumulative emissions exceeding 12.4 Gt CO_2_, as cheap credits delay fossil phase-out. Conversely, high carbon credit prices (200%–400%) discourage external credit use, leading to a sharp decline in fossil emissions and higher CDR scaling.

The cumulative emissions are lower (10 Gt CO_2_ at 400%), reaching a plateau around 2045 before reversing. The decreasing trend after 2046 means that LULUCF concepts are removing enough CO_2_, which enables us to use excess renewable carbon for synthetic fuels production and chemicals. The amount of biogenic carbon used in our model varies between 2.5 and 8.7 MtCO_2_ in a non-linear way across the different carbon credit scenarios implemented. While low-cost credits reduce total system expenditures (by 6% in the base case when compared to the most expensive), they jeopardize long-term climate targets driven by delaying fossil phase-out and the subsequent domestic CDR deployment. Therefore, prioritizing domestic CDR for durable removals and restricting credits to sectors with no viable alternatives could be a policy implication.

Furthermore, Figure S4 presents the detailed sensitivity analysis of BECCS toward multiple carbon credit price projections for interested readers. Our outcomes indicate that BECCS deployment is modest at low credit prices, with the model favoring cheaper external carbon purchases and less PtX utilization. However, as the credit prices increase, domestic CDRs become more cost-effective, leading to more CDR deployment and PtX uses as reliance shifts from external credits to domestic CDRs.

## Discussion

In global scenarios, it is almost universally assumed that both technical and natural sinks are required under ambitious climate targets.^9^ Yet, their combined economic and environmental implications in most countries’ decarbonization pathways have not been fully explored. Based on expert assessments of various CDR options, Borchers et al.^47^ estimate the theoretical maximum CO_2_ removal potential in Germany to be between approximately 91 and 240 Mt CO_2_/year by 2050. While these estimates provide valuable insights into the technical and scale-related capacities of different CDR strategies, they rely primarily on scenario-based and expert-informed assumptions that do not explicitly account for regional constraints, resource availability, and economic considerations. In this manuscript, we examine the optimal CO_2_ removal potential of integrating technology-based (BECCS) and natural-based CDRs within Germany’s bioenergy system, utilizing a spatially and politically detailed bottom-up optimization model—BENOPTex. Our model incorporates techno-economic and political factors of bio-based CDRs, such as biomass availability, land characteristics, and investment costs, to evaluate the feasibility of achieving climate targets. We explicitly account for the full spectrum of biomass resources, including agricultural residues (e.g., straw), forest residues, biowaste, digestates, and dedicated energy crops (e.g., miscanthus, sugar beet) with distinct roles in CDR strategies. Low-risk residues are prioritized for permanent carbon removal via BECCS (e.g., forest residues in combustion with CCS and gasification or biogas with CCS, which predominantly utilizes manure/slurry) due to their high scalability, lower cost, and carbon footprint (e.g., utilizing residues can avoid the emissions associated with their cultivation). Biowaste (e.g., organic digestates) and agricultural waste (e.g., straw) are routed to biochar production (PyCCS) for soil amendment, balancing carbon sequestration with circular economy benefits to meet biochar demand.

In addition to various residues, the model includes dedicated energy crops such as miscanthus and sugar beet, which play a significant role in scaling bio-based CDR over time. For example, miscanthus is allocated to combustion with CCS for heat provision, and sugar beet is the main feedstock for bioethanol production coupled with CCS. Furthermore, paludiculture biomass feeds mainly combustion plants with CCS. Our findings underscore the critical role of CDR in achieving climate targets. While rapid and deep emissions reductions remain paramount (avoidance strategies), the results demonstrate an increasing reliance on CDR technologies for achieving net neutrality and, more importantly, for achieving net-negative emissions (removing strategies). Even though the combination of BECCS and selected natural sinks can achieve approximately 110 Mt of CO_2_ removal by 2050 (This value could increase to ∼113 Mt CO_2_ when accounting for 3 Mt of carbon used for PtX and chemicals that remain in the system), a more diverse portfolio of CDRs is necessary to expand our options (e.g., long-lived materials in the construction sector, and set-aside concepts for other forest types) to meet Germany’s net-zero target more cost-optimally.

Among the natural sink options, TSA emerges as the most competitive across all scenarios, delivering around 38 MtCO_2_/year by 2050 at relatively low cost, particularly in the early decades when land availability is less constrained. While our model identifies TSA of beech forests as the most cost-effective natural CDR pathway, its deployment at such scale (2.39 Mha) is extremely challenging. The primary risk is carbon leakage; reducing domestic timber harvests could simply shift demand abroad, potentially leading to deforestation or forest degradation in other regions, thereby negating the global carbon benefit. A realistic CDR policy would therefore require stringent safeguards against such leakage, likely limiting the feasible scale of set-asides. Furthermore, this approach foregoes the potential for material-based CDR. Harvesting wood and using it in long-lived products (e.g., construction sector) also represents a form of carbon sequestration, while simultaneously displacing carbon-intensive materials such as concrete and steel. A more balanced strategy—one that combines limited, targeted set-asides in high-conservation-value areas with a strong focus on sustainable wood production for long-lived materials—may offer a more robust and sustainable path for the forestry sector’s contribution to climate mitigation. This strategy would secure carbon storage, reduce emissions via substitution, and maintain a vital domestic supply chain for a renewable resource. Therefore, the TSA potential presented in our results should be interpreted as an upper technical-economic bound under specific model assumptions, not a practical policy prescription. Future work must integrate these dynamic market interactions and sustainability constraints to define a truly feasible and sustainable portfolio for forest-based CDR.

Agroforestry delivers more modest but steady contributions of 6 Mt CO_2_/year depending on land conversion assumptions and ecological limits. Peatlands rewetting and paludiculture cultivation contribute around 5 MtCO_2_/year and show high potential in northern regions with large areas of drained organic soils. On the technological side, BECCS via combustion heat contributes 13–24 MtCO_2_/year, particularly in industrial applications and district heating. BECCS from biomethane proves to be among the most scalable and cost-efficient, reaching up to 30 MtCO_2_/year by 2050. It can benefit from existing infrastructure and biomass feedstock availability. BECCS for bioethanol production delivers up to 10 Mt CO_2_.

The technological CDR concepts become increasingly important as natural sinks approach saturation and land constraints tighten, while residual emissions stay in the system. This may also require the acquisition of carbon credits to offset residual emissions. Navigating the integration of external CDRs requires careful consideration of price and supply uncertainties, alongside the critical need to ensure it does not detract from ambitious fossil fuel emission reductions. The study shows that cheap international carbon credits delay fossil phase-out, while higher credit costs drive both deeper decarbonization and stronger domestic CDR deployment. This points to the importance of robust carbon market design. Moreover, we find that while the overall domestic CDR levels remain relatively stable across scenarios, the mitigation burden shifts between fossil reductions and offsetting depending on credit availability and pricing.

The modeling results gain even more relevance in light of recent political developments in Germany. CDR is being treated as an important component of Germany’s long-term climate strategy and residual-emissions management.^23^ The draft coalition agreement explicitly supports the deployment of “permanent and sustainable negative emissions” to address residual emissions and acknowledges that emissions reductions alone will not suffice to meet Germany’s 2040 climate targets. The agreement also enables the use of high-quality foreign carbon removal credits and pledges to integrate CDR into the EU ETS, a move that would create stable demand and investment signals across Europe.^48^

Our cost-optimal model results indicate a continued reliance on international carbon credits to offset emissions (with an estimated usage of approximately 70 Mt CO_2_ per year by 2045), when the carbon credit price is sufficiently low. This reflects a risk in carbon market design, where cheap offsets can disincentivize domestic decarbonization and CDR efforts. However, given our modeling assumptions, specifically the unlimited availability of credits at a low premium to domestic action, the credit volume is better interpreted as the scale of the ambition gap that must be addressed by other means. This gap underscores the critical need for deeper fossil emission reductions and to develop and deploy a more diverse portfolio of domestic CDR options beyond the bio-based methods studied here. This includes novel technical solutions such as DACCS^49^ and EW, to ensure Germany can meet its targets robustly and with high environmental integrity. A more politically realistic scenario, aligned with the emerging German and EU debate around strict limits and quality criteria for external credits, would involve a much higher effective price or a quantitative cap. As our sensitivity analysis shows (see Figure 7), such policies would shift the burden back onto deep domestic emission reductions and stimulate greater investment in CDRs.

These developments underscore the importance of the portfolio-based, spatially grounded CDR strategies explored in this study and suggest the need to better align policy ambition and implementation frameworks. Additionally, other aspects—such as the role of CO_2_ utilization within CDR systems (e.g., through PtX applications) or potential fossil fuel substitution—should neither be confused with CDR nor overlooked from a climate change mitigation perspective. This aligns well with the principles of a circular carbon economy, which emphasizes keeping carbon molecules in productive use rather than allowing them to accumulate in the atmosphere and avoiding extra storage costs. Besides infrastructure availability, economic incentives, such as carbon credits and pricing mechanisms, are key factors in determining whether the captured CO_2_ is stored or used for synthetic fuels and other industrial processes. While these exploitations do not constitute net-negative emissions, they play a vital role in defossilization, especially when combined with renewable electricity. Our modeling shows that integrating utilization pathways (PtX) into the broader CDR system design can enhance sectoral flexibility and optimize carbon flows across utilization and storage options.

Our sensitivity analysis of carbon prices and carbon credits depicts robust contributions from cheaper domestic bio-based CDR options, such as biomethane with CCS and heat combustion with CCS, while more expensive solutions (e.g., Gasification with CCS) may only be economically viable under extreme carbon pricing policies. Furthermore, we identified significant uncertainties in the analysis of natural sinks, particularly regarding the availability of land for conversion and utilization (e.g., forested areas, assuming the conversion of 1% of grasslands into forests). Therefore, policymakers should offer incentives to private grassland owners to support and encourage these conversions.^50^ The temporary nature of carbon storage in set-aside forests—particularly the complete preservation of 2.39 Mha of beech forests modeled in this study—introduces critical uncertainties for long-term CDR strategies and risks such as displacement effects due to leakage (the set-asides led to estimated leakage of 20 up to 100%).^51^ While this approach provides significant short-to-medium-term removal benefits, its long-term climate value hinges on post-2050 management decisions. Future work might assess permanence risks and develop governance frameworks to prevent carbon debt accumulation (e.g., through permanent protection or by redirecting harvested biomass into durable storage pathways such as biochar, BECCS, or long-lived materials).

Regional analysis reveals that the deployment of natural CDR options varies significantly depending on regional characteristics. While wetland-based approaches are more effective in the northern regions, forest-based solutions tend to be more successful in the south. These patterns underline the importance of place-based policy tools and land-use planning to support deployment.

This study can also be extended through comprehensive scenario analyses in the German context, considering the current state-of-the-art CDR technologies, relevant regulations, and stakeholder engagement. Moreover, incorporating other non-energy sectors, such as the food industry, can enhance our understanding of the interplay between human, ecological, and energy systems.

### Limitations of the study

This study has several limitations due to the complex nature of the problem:

1. Most energy system models analyze transitions up to 2050, reflecting widely adopted international climate policy targets.^52^^,^^53^^,^^54^ To ensure comparability with existing literature, this study therefore adopts 2050 as the end year of the analysis. Extending the analysis horizon beyond 2050 would introduce additional uncertainty into the results, particularly because several input datasets and supporting models used to generate key drivers, such as energy demand projections, are currently available only up to 2050. However, there is merit in extending the model’s temporal horizon to 2060 and beyond by incorporating the most recent scenario projections, as the wood extraction from TSA coincides with the end year of BENOPTex. Therefore, any CDR activity that sequesters carbon until 2050 is treated as “removed” for the duration of the analysis. The model does not account for carbon released after 2050, meaning that the “temporary” set-aside is emission-wise equivalent to the “permanent” set-aside within the model’s scope.
2. Endogenously modeling carbon leakage and the demand for timber are other shortcomings of this study. BENOPTex optimizes under techno-economic and regional biophysical constraints (land, biomass), but does not endogenously model non-energy market feedback, social acceptance, carbon leakage, permanence concerns for temporary storage, or other real-world barriers (However, we reduce the lost forest residues due to TSA/PSA from the available biomass for the energy sector). The model does not capture these carbon leakage dynamics associated with set-aside, and incorporating this would require coupling with a global forest trade model. That is why scientists call for better integration and understanding of earth and human systems to effectively capture cascades and telecoupling,^55^ as well as techno-economic CDR assessments^56^ to inform policymakers.
3. Also, the model does not explicitly account for the upstream infrastructure costs associated with CDR technologies, such as the construction and operation of CO_2_ pipelines and storage facilities. While these costs are implicitly considered within the overall technology investment costs, a more detailed analysis of these specific infrastructure expenses would provide a more comprehensive assessment. Detailed and accurate cost data availability is very limited due to the few pilot plants. In addition, cost estimates for natural sinks are highly context-specific, and only broad ranges/general estimates can be given.
4. The model ignores the potential risk of drought, forest fires, and forest pests and pathogens, which can negatively impact the carbon sequestration capacity of forest-based CDR approaches. Furthermore, emissions from land use and forests (beyond the selected concepts) are not explicitly accounted within the model’s framework, although they are negligible since 2010 see (https://climateactiontracker.org/countries/germany/); however, prolonged drought can transform forests from carbon sinks to carbon sources.^57^ In the analysis, agricultural emissions were also considered exogenous. The primary focus of our study was to model the removal of CO_2_ through bio-based CDR strategies (BECCS, forest management, soil management). The non-CO_2_ emissions from agriculture are complex (e.g., methane, nitrous oxide), involving biochemical processes that are not directly influenced by the CDR activities we modeled.
5. Similar to Sadr et al.,^28^ this study considers only the best yearly yields under the RCP2.6 scenario of the LPJmL model for energy crop yields, thereby neglecting more complex feedback mechanisms, such as temperature increases and changes in atmospheric CO_2_ concentration, which could affect the effectiveness of CDR strategies.
6. Finally, expanding the portfolio of CDR options under various scenarios. Although the analysis presented here, particularly the examination of a scenario without CDRs, provides valuable insights into the crucial role of direct emission reductions, it represents only one facet of the complex CDR landscape. Future work should investigate a wider range of CDR scenarios and options to fully understand their potential contributions, limitations, and interactions. Such analyses are essential for informing policy decisions related to CDR deployment and ensuring that it complements, rather than compromises, aggressive fossil emission reductions. For instance, future research can expand the CDR portfolio by incorporating the potential of biochar production and utilizing it in biochar-based building materials.

## Resource availability

### Lead contact

Further information and requests for resources and materials should be directed to and will be fulfilled by the lead contact, Mohammad Sadr (mohammad.sadr@ufz.de).

### Materials availability

This study did not generate new materials.

### Data and code availability

• Data: The input data used in this study, including regional biomass potentials, land-use data, and techno-economic parameters, are derived from publicly available sources and previous projects cited in the manuscript (e.g., Wollnik et al.^58^; Blickensdorfer et al.^39^^,^^59^). The input data specific to the scenario presented in this paper will be found at https://git.ufz.de/sadr/biocdr-in-germany-optimization. Processed data supporting the findings are available from the corresponding author upon reasonable request.
• Code: The core optimization model (BENOPTex) used in this study is open-source and available at https://git.ufz.de/sadr/biocdr-in-germany-optimization. The specific scripts for reproducing the scenarios presented in this paper are included in the same repository.
• Additional Information: Any additional information required to reanalyze the data reported in this paper is available from the lead contact upon request.

## Acknowledgments

The authors would like to express their sincere gratitude to Peter Elsasser, Ruben Seibert, and the anonymous reviewers for their feedback, which has significantly improved the quality of this manuscript. This work has been supported by the Federal Ministry of Research, Technology and Space of Germany (BMFTR) under the CDRterra project BioNET (grant no 01LS2107A). Also, Mohammad Sadr was partially funded by the *Man0EUvRE* (100695543), which is co-financed through taxation based on the budget adopted by the representatives of the Landtag of Saxony. “Man0EUvRE – Energy System Modeling for Transition to a net-Zero 2050 for EU via REPowerEU” is funded by CETPartnership, the European Partnership under Joint Call 2022 for research proposals, co-funded by the European Commission (GA N°101069750).

## Author contributions

M.S. conceptualization, writing – original draft, writing – review and editing, visualization, validation, software, methodology, formal analysis, and data curation. D.E.A. conceptualization, methodology, software, supervision, and writing – original draft. M.J. methodology, software, and data curation. R.W. data curation. D.T. writing – original draft, resources, and supervision.

## Declaration of interests

The authors declare that they have no competing interests.

## STAR★Methods

### Key resources table

**Table:** 

REAGENT or RESOURCE	SOURCE	IDENTIFIER
**Deposited data**		

Techno-economic parameters for CDR options	Wollnik et al.^58^	https://doi.org/10.48480/x293-8050
German forest species composition data	Blickensdorfer et al.^59^	https://doi.org/10.3220/DATA20221214084846
German agricultural land-use maps	Blickensdorfer et al.^39^	https://doi.org/10.5281/zenodo.15309479

**Software and algorithms**		

BENOPTex optimization model	Helmholtz Center for Environmental Research - UFZ	https://git.ufz.de/sadr/biocdr-in-germany-optimization
MATLAB (R2020a or later)	MathWorks	https://www.mathworks.com/products/matlab.html
GAMS (General Algebraic Modeling System) v32.2.0 with CPLEX	GAMS Development Corporation	https://www.gams.com

**Other**		

German Climate Action Law	Federal Ministry for the Environment, Nature Conservation and Nuclear Safety -- BMUV	https://www.gesetze-im-internet.de/englisch_ksg/englisch_ksg.pdf
LPJmL model (RCP2.6 scenario yields)	Frieler et al.^60^	https://gmd.copernicus.org/articles/10/4321/2017/gmd-10-4321-2017.pdf

### Method details

The main objective of this study is to evaluate the cost-optimal deployment of CDRs by combining BECCS and natural sinks and integrating them into an already established bioenergy system model to achieve Germany’s net-zero and subsequently negative emission targets. To achieve this, we first incorporate various CDR pathways (i.e., 6 BECCS technologies, 6 forest-based options, 6 agriculture practices, and two concepts of peatland rewetting and paludiculutre cultivation), as well as region-specific data (e.g., dedicated energy crop cultivation by type and quantity, and the area covered by various forest types and agricultural lands) into our energy system model. Then, by using modeling and optimization, we determine the most effective combinations of CDRs as well as their optimal timing and deployment scale to achieve climate targets. Lastly, we assess the potential contribution of CO_2_ removal for each option in Germany as well as the trade-offs and synergies between various choices through sector-coupling.

#### Data description and assumptions

The primary techno-economic source for this study is Wollnik et al.,^58^ wherein the authors evaluated a variety of CDRs (including BECCS and natural sinks) in standardized fact sheets that reported in detail the costs, energy and material requirements, outputs, and potential for CO_2_ removal. Tables S3 and S4 provides techno-economic details of selected natural sink concepts for this study. Our CDR portfolio consists of four clusters, including BECCS, forest management, agriculture, and soil practices, as well as peatland rewetting and paludiculture, as illustrated in Figure S1. Although our analysis take all these concepts into account, we focus more on explaining natural sinks here and referring interested readers to^28^ for detailed information on BEECS.

The set-aside calls for temporarily or permanently abandoning the forests. The beech forest is the only area in this study where the *“set-aside”* concepts can be applied. This is due to the fact that their growth characteristics enable them to accumulate high carbon stocks and store them longer than the usual rotation period. Furthermore, Germany has a large number of beech trees, about half of which are over a century old.^58^ There are, however, conflicts of use arising from the set-aside concept, since the wood remains in the forest and is not available for sale or use for material purposes. Afforestation, on the other hand, involves establishing forests on land that was not previously used for forestry, such as agricultural land, to act as carbon sinks. This is achieved by planting seedlings, which enables long-term carbon sequestration. Germany does not have a specific quantitative target or policy for implementing a certain area of afforestation within the country. However, extreme weather events like droughts and bark beetle infestations have caused damage, leading to the need for afforestation on about 277,000 ha of land by mid-century.^61^ Germany’s Federal Ministry of Food and Agriculture (BMEL) also stated that the forest area in Germany has increased by more than 200,000 ha since 1990. Grassland is a plausible choice to be employed for afforestation purposes. Based on expert assessment, we projected 50,000 ha of afforestation land (less than 1% of the total grassland areas in Germany) for the afforestation of beech, Douglas fir, oaks, and Scots pine in the study until 2050.

Details and spatially explicit information about the species composition of trees in forests are gathered from Blickensdörfer et al.^59^ and Welle et al.^40^ In Blickensdörfer et al.,^39^ comprehensive maps of the agricultural landscapes in GeoTiff for the years 2017, 2018, and 2019 are offered. To overcome periods of heavy cloud contamination, this dataset merged monthly composites of Sentinel-1 backscatter data with densely interpolated time series of Landsat and Sentinel-2A/B data. The model’s accuracy has been documented in.^39^ We used these data in our study to determine the available land covered by various natural sinks in the country for each state, based on regional characteristics.

All-year ground cover is a method where the soil is consistently covered with plants, which increases carbon input and storage in the soil and avoids the issue of fallow arable land. We used catch crop as the proxy of *“all year ground cover”* option to estimate the potential available land and growth rate for its implementation. In 2019/2020, catch crops were cultivated on a total area of 1,974,000 ha of arable land compared to 1,189,000 ha in 2009/2010. We limited these options to be applied to a maximum of 10% of agricultural lands (1.84 Mha) in the model.^62^ Incorporating organic fertilizers and compost into the soil increases the carbon content through the applied materials and improves the soil’s physical properties and nutrient availability. This soil improvement enhances plant productivity, resulting in increased carbon accumulation in the soil by the plants. Cultivated area for organic farming data (only organic fertilizer is used here as a representative of the concept of *“organic fertilizer and compost”*) linearly interpolated between 1995 and 2023 to find the annual increase in the country, starting from 309,487 ha in 1995 and reaching 1,869,227 ha in 2023. This is equivalent to a maximum of 55,705 ha per year. In no-till practices, crops are sown and harvested simultaneously using a no-till machine. This decreases tillage and soil erosion. Additionally, the organic carbon stored in the soil is protected, and the continuous soil cover by the crops and their roots contributes extra carbon to the soil. *“No-till”* and *“conversion of arable land into permanent grassland”* concepts, on the other hand, are not widespread in Germany and are limited to growing. According to the Federal Statistical Office, only 93,900 ha were cultivated with no-till farming in 2015/2016. However, we allow the model to select these options by considering 5% of its current area annually.^63^ The technical details of all agricultural options can be found in Table S4. Agroforestry systems involve the integration of woody plants like trees, hedges, and shrubs with agricultural land. This combination leads to higher carbon accumulation in the soil. In this study, we also assumed that a maximum of 10% of grassland (510,570 ha) is available to use for *“agroforestry”* option. Teichmann^64^ assesses the climate protection through biochar in German agriculture. They found that the greatest biochar potentials are from liquid manure, digestates, and forestry residues in 2030. With an average assumption of 1.5 t of biochar application in each hectare of agricultural land, and using the forestry residues amount we derived the potential growth rate for this option.

Rewetting drained peatlands involves restoring a stable water table near the surface. This can immediately reduce or stop net carbon loss and may also lead to new carbon sequestration in the litter layer and peat. Carbon is stored permanently as long as the peatland remains wet. In Gerrits et al.,^65^ the annual rewetting is reported as 50,000 ha; however, the current speed is less than 2000 ha/yr. To set the growth rate in the model, we interpolate between these two values from 2020 to 2050. Paludiculture is a productive land use approach on wet and rewetted peatlands. It includes various forms of biomass use, from harvesting spontaneous vegetation on semi-natural sites to growing newly established crops on rewetted sites, under conditions that preserve the peat body or encourage new peat accumulation.

The central carbon price pathway rises from €20/tCO_2_ in €2020 to €250/tCO_2_ by 2050. This trajectory is aligned with mid-range estimates from decarbonization scenarios for advanced economies (e.g., Moore et al.,^66^ Günther et al;^67^), representing a plausible incentive level needed to drive deep decarbonization and CDR deployment. The price of international carbon credits in the base case is set at a 10% premium to the domestic carbon price. This premium is a modeling choice reflecting a policy intent to prioritize domestic investment, and its impact is thoroughly tested in a sensitivity analysis where the premium is varied from 50% to +400% (Section the impact of carbon credit).

This study focuses exclusively on the base scenario, in which net neutrality by 2045 is the target. Through the cost-effective use of BECCS and natural-sink options in our portfolio, we assess their potential to support Germany’s total GHG budget to remain within the specified limits.

#### Model specifications and description: BENOPTex

To accomplish the techno-economic analysis of CDRs, cost-effectively achieving climate targets, while utilizing shared resources, we employ an optimization model, the so-called “BENOPTex”.^30^ BENOPTex is a deterministic linear optimization with perfect foresight model, developed to optimally allocate biomass and dispatchable renewable resources in the power, heat, transport, and chemical sectors. It utilizes exogenous input databases, including techno-economic and political parameters, resource potentials, prices, and scenario-specific drivers and constraints. An interface developed in MATLAB imports the required data from external datasets and performs pre-processing computations, such as calculating the annualized investment costs, interpolating feedstock prices, assessing the excess electricity, and computing the life cycle GHG emissions. Subsequently, the processed data is transferred to the back-end model, which is implemented in GAMS (General Algebraic Modeling System) for solving the optimization problem. The generated model, based on provided data, is solved using CPLEX solver, to optimally allocate limited resources–such as biomass, available land, and other imported energy carriers such as PtX through technologies–to satisfy demands across sectors. The allocation is optimized to minimize costs while adhering to constraints such as capacity expansion limits, primary resource availability, GHG-specific emission targets, and sectoral demands to be fulfilled.

To enhance the computation speed, we allow the model to have flexible resolution in multiple dimensions, including temporal, spatial, technological, and sectoral, while employing multiple solution methods (i.e., interior point and primal and dual simplex) in parallel.^68^ For instance, to evaluate the effect of seasonality on bioenergy production in Germany, a daily resolution was implemented to evaluate the availability of energy crops.^69^ In spite of the fact that the mitigation benefits of CDRs are global, risk, costs, and environmental impacts (or cobenefits) may be geographically localized.^11^ Therefore, we choose to gather GIS information to represent all German states in this effort. We study the possible regional deployment of our CDR portfolio, leading to improved CO_2_ removal potential estimations, by increasing the spatial resolution of the optimization model to 16 states in Germany, which is comparable to NUTS-1 (i.e., Nomenclature of territorial units for statistics). Hence, the model benefits not only from a high technological resolution but also from temporal and spatial resolution. This approach has also been extended to other renewable energy sources outside the model boundary. For instance, in the case of wind energy, we rely on high-resolution simulation results from the BAU scenario presented in Aliabadi et al.^70^

It is also necessary to address intangible aspects of CDRs, such as social acceptance, secondary impacts, co-benefits, infrastructure requirements, adverse side effects, and trade-offs (e.g., biodiversity loss or water resources); nonetheless, these fall outside the scope of our modeling.

We minimize total system costs^28^ while considering specific GHG targets over different sectors across the entire planning time horizon (i.e., 2020–2050). Using the total emission quantities permitted in the various sectors pursuant to the Federal Climate Change Act,^23^ we set the RCB over the years until we reach carbon neutrality in 2045 and negative emissions thereafter. In this manuscript, our focus is only on constraints that directly affect natural-sink options. Using a regionalized bottom-up potential assessment of BECCS in Germany, we examined solely six BECCS pathways in detail in Sadr et al.^28^ Additionally, the interested reader can see^28^^,^^29^^,^^71^ where other constraints are well documented. Here, we build upon Sadr et al.^28^ to analyze how the combined technical sinks and natural sinks can interplay and affect Germany’s potential to meet climate targets. Equation 1 defines the total annual cost of implementing *natural-sink* concept *i*, at year *t* in region *r*, where *C*_*tir*_ and *M*_*tir*_ depict the annualized capital and marginal cost per unit of land area, respectively. The decision variable ***k***_*tir*_ indicates the quantity of land installed in hectares over time for each concept in regions. Depending on the region, we considered the marginal cost of land for various afforestation options. However, the cost of land is excluded for the set-aside options, but the opportunity cost – such as the cost of wood is considered in the cost calculation. Moreover, to account for opportunity costs that must be added to the CAPEX associated with *”converting arable land to grassland”*, we employed the price of wheat as a proxy. This equation adds to the main objective function where the total system cost is introduced (see Objective function & constraints).

(Equation 1) $$\begin{array}{cc} z_{t}^{\text{NS}}=\sum_{i,r}\left(C_{tir}+M_{tir}\right)\times k_{tir} & \forall t\in T \end{array}$$

We expand the GHG budget equation from Sadr et al.^28^ (see Objective function & constraints) to combine the potential removal of BECCS and natural sinks. This led to the LULUCF removal amount contributing to the total country’s GHG budget in addition to BECCS from 2040 onward. Therefore, Eq. (2) supplements Eq. (8). It determines the amount of sequestrated CO_2_ at year *t* by the negative emission concept *i* in region *r*, where $\varepsilon_{tir}^{-}$ is the negative emission factor of option *i* per unit of land area, and $\psi_{t,i,r}^{\text{NS}}$ is a decision variable that specifies the amount of CO_2_ sequestrated by natural-sink *i* in year *t*, in various regions. The set of forest-based, agriculture, and peatland rewetting options is represented by *I*^*F*^, *I*^*A*^, and *I*^*P*^, respectively. We assumed emission factors to be similar across all regions, while they vary over the lifetimes of each option.

Equation 3 establishes a minimum limit on negative GHG emissions in natural sinks, taking into account the stated targets in the LULUCF sector (i.e., $\tau_{t}^{\text{LULUCF}}$) in million tonnes. For the intermediate years between 2020 and 2050, the LULUCF targets are interpolated. To avoid infeasibility, an additional decision variable is introduced, ${\hat{p}}_{t}^{{\text{CO}}_{2}}$, which expands the feasible space by allowing the acquired CO_2_ credit from outside Germany to compensate for the overshoot from the stated targets. In order to prioritize using domestic resources to meet climate targets, we assume the carbon credits price from outside will cost 10% more than the carbon price in the same year. We include this in Equation 7 to reflect that into our objective function. Equation 4 assumes that natural-sink concepts will grow over time and cannot shrink.

(Equation 2) $$\varepsilon_{\mathrm{tir}}^{-}\times k_{\mathrm{tir}}=\psi_{\mathrm{tir}}^{\text{NS}}\forall t\in T,i\in I^{\mathrm{NS}}=I^{F}\cup I^{A}\cup I^{P},r\in R$$

(Equation 3) $$\underset{i,r}{\sum }\frac{\psi_{\mathrm{tir}}^{\text{NS}}}{10^{6}}+{\hat{p}}_{t}^{{\text{CO}}_{2}}\geq \tau_{t}^{\text{LULUCF}}\forall t\in T$$

(Equation 4) $$k_{\mathrm{tir}}\geq k_{t-1,ir}\forall t\in T,i\in I^{\mathrm{NS}},r\in R$$

(Equation 5), (Equation 6) set an upper bound for the capacity expansion of natural sinks in the study. The ${\bar{K}}_{\mathrm{tir}}$ illustrates the maximum expansion rate in each region, for the various concepts over time (i.e., afforestation of Beech, Douglas fir, Oak and Scots Pine and agriculture options of All-year ground cover, Organic fertilizer and compost, no-till, conversion of arable land to grassland, agroforestry, and biochar application) while because of competition for the same forest beech area, this rate for the *“set-aside”* (SA) of beech forests (i.e., permanent set-aside and temporary set-aside) is expressed separately. When expanding capacity, we assume no change in the distribution shape of forest types and agricultural lands in each region by taking their relative amounts in each region and comparing them to their overall area in the country to determine the upper bound ${\bar{K}}_{\mathrm{tir}}$.

(Equation 5) $$k_{t,i,r}-k_{t-1,i,r}\leq {\bar{K}}_{t,i,r}\forall t\in T,r\in R,i\in I^{NS}\left\{I^{\mathrm{SA}}\right\}$$

(Equation 6) $$\underset{i\in I^{SA}}{\sum }\left(k_{t,i,r}-k_{t-1,i,r}\right)\leq \frac{\sum_{i\in I^{SA}}{\bar{K}}_{t,i,r}}{2}\forall t\in T,r\in R$$

We have adjusted the available peatland use for energy purposes in BECCS and other technologies. This factor, which was previously an external parameter,^28^ is now integrated as a decision variable in our model as illustrated in Equation 18. Since this variable can impact the removal potential of BECCS technologies, especially combustion plants with CCS for electricity generation, we will perform a sensitivity analysis on it later. Also, additional equations implemented into the model are available in Objective function & constraints, (Equation 11), (Equation 12), (Equation 13), (Equation 14), (Equation 15), (Equation 16), (Equation 17), (Equation 18), (Equation 19), (Equation 20), (Equation 21), (Equation 22) for interested readers.

#### Objective function & constraints

As mentioned in Section method details, the objective function minimizes the total system cost, which is presented in a stylized form by Equation 7.

(Equation 7) $$\begin{array}{ll} \text{min}\;z^{c} & =\overset{z_{1}}{\overset{︷}{\text{Production}\;\text{costs}}}+\overset{z_{2}}{\overset{︷}{\text{Investment}\;\text{costs}}}\\ & \;+\underset{z_{3}}{\underset{︸}{\text{Domestic}\;\text{feedstock}\;\text{costs}}}\\ & \;+\overset{z_{4}}{\overset{︷}{\text{Imported}\;\text{feedstock/synthetic}\;\text{fuel}}}\\ & \;+\overset{z_{5}}{\overset{︷}{\text{Imported}\;\text{carbon}\;\text{credit}\;\text{cost}\;}}\\ & \;-\overset{z_{6}}{\overset{︷}{\text{Revenue}\;\text{from}{\text{CO}}_{2}\text{storage}}}\\ & \;-\underset{z_{7}}{\underset{︸}{\text{Linearizing}\;\text{term}}}+\underset{z^{NS}}{\underset{︸}{\text{Natural}\;\text{sink}\;\text{costs}}} \end{array}$$

The objective function *z*^*c*^ minimizes the total system cost. *z*^*c*^ consists of the following terms: The production costs, (*z*_1_), investment costs (*z*_2_), consumed feedstock/fuel (including domestic *z*_3_ and imported *z*_4_), the total cost of compensating using external credits (*z*_5_), revenue from selling credit for sequestrating carbon (*z*_6_), penalties (*z*_7_) to linearize GHG quota constraints, and forest management costs. To be in line with REPowerEU,^72^ we assume that imported synthetic fuel is more expensive than domestically produced fuel, considering external costs such as reliability and sustainability. The detailed explanation is presented in Aliabadi et al.^71^ and Sadr et al.^28^

Equation 8 determines the annual net emission $\left(B_{t}^{{\text{CO}}_{2}}\right)$ within the model, which is influenced by the fossil-based sources that persist in the system and the achievable amount of CDR. The national net emission is also pursued via the economic incentive of an exogenously set carbon price. This approach allows the model to identify cost-optimal pathways that may involve a residual net emissions gap if offset by domestic emission reductions (e.g., renewables which replace fossil sources), domestic CDR or international carbon credits (purchased CDR). In Equation 8, $\varepsilon_{ti}^{FF}$ is the emission factor of fossil fuels consumed by technology *i* at year *t* (in kt CO_2_eq/PJ) and $\delta_{\mathrm{dti}}^{FF}$ represents the total amount of fossil fuels used by technology *i* at time slice *d* of year *t* in PJ. The second and third terms represent the removal potential of chemicals and BECCS, respectively. Within the model, $m_{t}^{{\text{CO}}_{2}used}$ denotes the captured biogenic CO_2_, an output of bioenergy processes that can be directed toward utilization in PtX synthesis or chemicals (i.e., methanol). The allocation is determined by the model’s cost optimization. CO_2_ directed to storage is classified as CDR, generating permanent negative emissions, while CO_2_ directed to PtX or chemicals is treated as a circular carbon flow. This modeling choice allows us to show a key finding that biogenic CO_2_ is a valuable resource. There is a trade-off between using it for permanent CDR and using it to defossilize sectors like aviation and shipping via PtX.

The model is constrained to meet Germany’s legislated annual sectoral emission budgets ${\bar{B}}_{t}$ as a hard constraint, as demonstrated in Figure 1 and outlined in Equation 9. In this constraint, $\varepsilon_{t}^{AG}$ denotes emissions from non-energy sectors (e.g., agriculture). $m_{t}^{{\text{CO}}_{2}}$ determines the amount of CO_2_ purchased from other countries in order to compensate for the amount that is above the declared targets. CO_2_ credit price plays an important role in the economic viability of BECCS technologies. The higher the CO_2_ credit price, the higher the contribution of domestic BECCS concepts in CO_2_ removal. We assume that ${\hat{p}}_{t}^{{\text{CO}}_{2}}$ is slightly higher (10% more expensive under the base scenario) than the carbon price $\left(p_{t}^{{\text{CO}}_{2}}\right)$ in the same year; therefore, our model tries to reach climate targets using domestic resources. We add this cost to the main objective function to reflect that (see Equation 7). A critical physical and infrastructural limitation considered in the model is the availability of geological storage sites for captured CO_2_. This is implemented via a dynamic storage capacity constraint, which ensures that the total amount of CO_2_ stored in any given year cannot exceed the available capacity $V_{t}^{{\text{CO}}_{2}}$, as it is formalized in Equation 10.

(Equation 8) $$\underset{d,i}{\sum }\left(\varepsilon_{ti}^{FF}\times \delta_{\mathrm{dti}}^{FF}\right)-\underset{i}{\sum }\left(\varepsilon_{ti}^{-}\times \pi_{\mathrm{tis}}^{ch}\right)-\underset{i}{\sum }\psi_{ti}^{BECCS}-\underset{i,r}{\sum }\psi_{\mathrm{tir}}^{\text{NS}}+m_{t}^{{\text{CO}}_{2}used}=B_{t}^{{\text{CO}}_{2}}\forall t\in T$$

(Equation 9) $$\frac{B_{t}^{{\text{CO}}_{2}}}{1000}+\varepsilon_{t}^{AG}-\frac{m_{t}^{{\text{CO}}_{2}}}{1000}\leq {\bar{B}}_{t}\forall t\in T$$

(Equation 10) $$m_{t}^{{\text{CO}}_{2}stored}=\underset{i}{\sum }\psi_{ti}^{BECCS}-m_{t}^{{\text{CO}}_{2}used}\leq V_{t}^{{\text{CO}}_{2}}\;\forall t\in T$$

Equation 11 establishes an upper limit for two *set-aside* options in 2050, which should not be greater than $\Lambda_{\mathrm{2020,r}}^{\text{Beech}}$, the total amount of land accessible from the beech forests in the regions. This amount of setting aside rests on a simplifying assumption that domestic wood demand adjusts proportionally to reduced supply, with no increase in imports or harvesting elsewhere. In reality, a large-scale set-aside would likely trigger carbon leakage through increased timber imports, potentially offsetting a significant portion of the domestic carbon gain. This maximum amount likewise limits each of the four *forestry* options represented by $\Lambda_{\mathrm{2020,r}}^{\text{Grassland}}$ in region *r*, allocating 10% of that region’s grassland as shown in Equation 12.

(Equation 11) $$\underset{i\in I^{SA}}{\sum }k_{\mathrm{2050,i,r}}\leq \Lambda_{\mathrm{2020,r}}^{\text{Beech}}\forall r\in R$$

(Equation 12) $$\underset{i\in I^{\mathrm{Plantation}}}{\sum }k_{\mathrm{2050,i,r}}\leq 0.1\times \Lambda_{\mathrm{2020,r}}^{\text{Grassland}}\forall r\in R$$

Equation 13 constraints the amount of land $\Lambda_{\mathrm{2020,r}}^{\text{Grassland}}$, originated from grassland in region *r* that can be converted to *“agroforestry”* by 2050 in that region. Likewise, the amount of agricultural land in each region that *“All year ground cover”* concept can be applied to by 2050 is restricted in Equation 14.

(Equation 13) $$k_{\mathrm{2050,i=Agroforestry,r}}\leq 0.1\times \Lambda_{\mathrm{2020,r}}^{\text{Grassland}}\forall r\in R$$

(Equation 14) $$k_{\mathrm{2050,i=AGC,r}}\leq 0.1\times \Lambda_{\mathrm{2020,r}}^{\text{Agriculture}}\forall r\in R$$

In Equation 15, the decision variable ***k***_*2050,i=Rewetting,r*_ represents the hectare of land that has been rewetted or transformed into *“wetland”* by 2050. For the conversion, the maximum amount of land that may be used was assumed to be 5% of each region’s grassland and agricultural area. Equation 16 establishes a subtarget for the country’s rewetted CO_2_ removal^73^^,^^74^ in order to support the achievement of the climate targets by generating negative emissions from wetland. In this equation, ***ψ***_*2030,i=Rewetting,r*_ represents the regional rewetting-based carbon removal in 2030 (in tonnes of CO_2_). The $g^{{\text{CO}}_{2}}$ term is a dummy variable to prevent infeasibility in the solution space. (17) defines the initial wetland area in the model. Assuming an average wetland area of 2,000 ha from 1980, we estimate the 2020 wetland area to be 80,000 ha.^75^

(Equation 15) $$k_{\mathrm{2050,i=Rewetting,r}}\leq 0.05\times \left(\Lambda_{\mathrm{2020,r}}^{\text{Agriculture}}+\Lambda_{\mathrm{2020,r}}^{\text{Grassland}}\right)\forall r\in R$$

(Equation 16) $$\underset{r}{\sum }\psi_{\mathrm{2030,i=Rewetting,r}}+g^{{\text{CO}}_{2}}\geq 5,000,000$$

(Equation 17) $$\underset{r}{\sum }k_{\mathrm{2020,i=Rewetting,r}}=80,000$$

Equation 18 connects the energy and agriculture sectors in the model. ${\dot{m}}_{t,i,f,c,r}$ stands for the amount of feedstock in *PJ* that technology *i* uses to generate electricity, heat, biofuels, or biochemicals in a variety of regions over the time *t*. The land demand for crop *f* in region *r* is denoted by parameter *Y*_*t*,*f*,*r*_ in $\frac{ha}{PJ}$. The maximum wetland area in each region $\Lambda_{\mathrm{2020,r}}^{\text{Wetland}}$ limits the amount of land needed for cultivation of energy crops (i.e., paludiculutre). 95% of the country’s wetland is drained for various uses,^58^ and we assumed a linear increase in paludiculture cultivation for energy purposes, from 2% in 2020 to 20% by 2050, represented by *p*_*t*_. Equation 19 ensures that the total utilization of a specific regionalized forest residue by all sectors (power, transportation, heat, and chemicals) in a given time period does not exceed the available potential of that residue within the region. This constraint helps to ensure the sustainability and realism of the model by limiting the use of forest residues to available resources. The term ${\bar{R}}_{t,f,r}$ denotes the total available residues in the regions (in this case, limited to the forest residues), while the second term of the left hand side accounts for the portion of forest residues that are set aside for purposes other than energy and chemical uses. Since the term ***k***_*t*,*i*,*r*_ is in *ha*, it needs to be converted to *PJ* using the conversion factor *ω*.

(Equation 18) $$\underset{i,f=Paludi,c}{\sum }Y_{t,f,r}\times {\dot{m}}_{t,i,f,c,r}\leq p_{t}\times \Lambda_{\mathrm{2020,r}}^{\text{Wetland}}\forall t\in T,r\in R$$

(Equation 19) $$\underset{r}{\sum }{\bar{R}}_{t,f,r}-\underset{i,r}{\sum }k_{t,i,r}\times \underset{\left(\frac{t}{ha}\times \frac{PJ}{t}\right)}{\underset{︸}{\omega }}\geq \underset{i,f,c,r}{\sum }{\dot{m}}_{t,i,f,c,r}i\in I^{\mathrm{SA}},f=\text{forest}-\text{residues},\forall t\in T$$

Equation 20 determines the quantity of digestates produced $\pi_{t}^{\mathrm{digest}}$ in *PJ* over time. In this equation *β*_*i*_ is the ratio of the byproduct in $\frac{tDM}{GJ}$ to the main output of the technology *i* in the set of technologies producing digestates *I*^*digest*^, and ***π***_*t*,*i*,*s*_ is the main output from technology *i* at time *t* for sector *s*. Digestates are a byproduct of several technologies in the model, such as BECCS_biogas, BECCS_biomethane, BECCS_ethanol, BME (biomethane based on anaerobic digestion), and PBME (power-to-methane via biological methanation). Lower heating value of digestates is referred to as *σ*^*digest*^ in $\frac{GJ}{tDM}$. Figure S2 illustrates the cascade use of biomass to produce biochar using digestates, a byproduct of some biomass technologies, and the possible demand for it (we haven’t yet taken the demand in the construction sector).

Equation 21 states that the total biochar production at time *t* is calculated as the sum of two components: 1- Digestate-based biochar, 2- Other feedstock-based biochar. The first term, represents the amount of biochar produced from digestates at time *t*. It’s calculated by multiplying the total digestates produced $\pi_{t}^{\mathrm{digest}}$ by an efficiency factor *η*_*t*,*i*=*PyCCS*_ associated with the *PyCCS* (pyrolysis of waste, straw and residues) technology. The second term calculates the biochar production from various feedstock types. It sums up the products of different feedstock types ${\dot{m}}_{t,i,f,c,r}$ and their respective conversion efficiencies *η*_*t*,*f*,*i*=*PyCCS*_ across all relevant indices mentioned before.

(Equation 20) $$\pi_{t}^{\mathrm{digest}}=\sigma^{\mathrm{digest}}\times \underset{i\in I^{\mathrm{digest}},s}{\sum }\beta_{i}\times \pi_{t,i,s}\forall t\in T$$

(Equation 21) $$\pi_{t}^{\mathrm{biochar}}=\pi_{t}^{\mathrm{digest}}\times \eta_{t,i=PyCCS}+\underset{f,c,r}{\sum }{\dot{m}}_{t,i,f,c,r}\times \eta_{t,f,i=PyCCS}\forall t\in T$$

(Equation 22) $$\pi_{t}^{\mathrm{biochar}}\geq D_{t,s=biochar}\forall t\in T$$

Equation 22 shows that the biochar demand ***D***_*t*,*s*=*biochar*_ should be satisfied by the amount of biochar produced $\pi_{t}^{\mathrm{biochar}}$ at year *t*.

Figure S2 illustrates the flow and utilization of biogenic resources within the BENOPTex model, particularly focusing on how these resources are processed and converted into various outputs, including energy, fuels, and chemicals. Biogenic resources such as residues, energy crops, and peatland are processed through various conversion pathways coupled with carbon capture and storage (CCS) to reduce emissions. Captured CO_2_ from bioenergy processes is stored underground or used in industrial applications (CCU), contributing to negative emissions. As exhibited in Figure S2, cascading use of biomass, where biogenic resources are utilized in multiple stages, maximizes obtained values while minimizing waste by prioritizing biomass usage in various applications. For instance, biomass is first used for high-value applications (e.g., biofuels, chemicals). Any remaining residues are then used for lower-value applications (e.g., digestates for biochar production). The blue arcs represent the cascade use of biomass identified in our study. For example, using sugar beet, bioethanol with CCS technology produces bioethanol, which is partially used in chemical industries to produce Ethylene, a feedstock for the production of polymers thereafter. Furthermore, some of the sequestrated CO_2_ is converted to synthetic fuels (PtX), as renewable CO_2_ holds significant value in energy and non-energy sectors.^76^

The data related to the transmission network is taken from the GENeSYS-MOD model.^77^ The flow of electricity between regions should not exceed the capacity of the transmission lines. Also, the projections of regional electricity demand and hourly patterns are adopted from the Renewable Energy Mix (REMix) model.^52^ The flows of electricity between regions are governed via (Equation 23), (Equation 24). Equation 23 sets a cap $\left({\hat{F}}_{t,r_{1},r_{2}}\right)$ for electricity flow between two regions *r*_1_ and *r*_2_. In Equation 24, electricity used at time slice *d* of year *t* in region *r* (i.e., ${\dot{m}}_{\mathrm{dtr}}^{el}$) should be less than the domestic excess production of electricity in region *r* (i.e., $\pi_{\mathrm{dtr}}^{el}-\delta_{\mathrm{dtr}}^{el}$), net electricity dispatched from other regions to region *r* and imported electricity to region *r* (i.e., $\pi_{\mathrm{dtr}}^{el-imp}$) combined, considering the loss in the network (0.97% in each 1000 km). |*D*| is the total number of times slices.

(Equation 23) $$F_{t,r_{1},r_{2}}+F_{t,r_{2},r_{1}}\leq {\hat{F}}_{t,r_{1},r_{2}},\forall t\in T,r_{1},r_{2}\in R$$

(Equation 24) $${\dot{m}}_{\mathrm{dtr}}^{el}\leq \left(\pi_{\mathrm{dtr}}^{el}-\delta_{\mathrm{dtr}}^{el}\right)+\frac{\sum_{r_{2}}\left(\left(0.97^{\left\|r-r_{2}\right\|}F_{t,r_{2},r}\right)-F_{t,r,r_{2}}\right)}{\left|D\right|}+\pi_{\mathrm{dtr}}^{el-imp},\forall t\in T,d\in D,r,r_{2}\in R$$

(Equation 25), (Equation 26) define the *average levelized cost of removal (aLCOR)* calculated over the modeling horizon *t*. The numerator in each expression is the cost attributed to the CDR technologies, for BECCS in the first scenario, and for the combined BECCS and natural sinks in the second scenario. The denominator is the present-valued total removed CO_2_ over the same horizon. All cash flows and removals are discounted by the factor (1 + *d*)^*t*^ to produce a single, comparable levelized cost in €/tCO_2_.

(Equation 25) $$aLCOR^{BECCS}=\frac{\sum_{t=2020}^{T}\frac{\sum_{i,r}^{I,R}C_{t,i,r}^{BECCS}}{{\left(1+d\right)}^{t}}}{\sum_{t=2020}^{T}\frac{{\sum_{i,r}^{I,R}\psi }_{i,r}^{\text{BECCS}}}{{\left(1+d\right)}^{t}}}t\in T,i\in I^{\mathrm{BECCS}},r\in R$$

(Equation 26) $$aLCOR^{\left(BECCS+NS\right)}=\frac{\sum_{t=2020}^{T}\frac{\sum_{i,r}^{I,R}\left(C_{t,i,r}^{BECCS}+C_{t,i,r}^{NS}\right)}{{\left(1+d\right)}^{t}}}{\sum_{t=2020}^{T}\frac{\sum_{i,r}^{I,R}\left(\psi_{i,r}^{BECCS}+\psi_{i,r}^{NS}\right)}{{\left(1+d\right)}^{t}}}t\in T,i\in \left(I^{\mathrm{BECCS}}\cup I^{\mathrm{NS}}\right),r\in R$$

(Equation 27), (Equation 28) define *removal intensity* (*RI*_*t*_) for each year *t*, expressed as tCO_2_ per hectare. For each region *r*, the removal intensity is calculated as the total removal achieved by the land-using technologies divided by the land area allocated to them in that year.

(Equation 27) $$RI_{t}^{\text{BECCS}}=\frac{\sum_{i,r}\psi_{i,r}^{\text{BECCS}}}{\sum_{i,r}A_{i,r}}i\in I^{\mathrm{BECCS}},r\in R,\forall t\in T$$

(Equation 28) $$RI_{t}^{\left(\text{BECCS}\;+\;\text{NS}\right)}=\frac{\sum_{i,r}\left(\psi_{i,r}^{\text{BECCS}}+\psi_{i,r}^{NS}\right)}{\sum_{i,r}A_{i,r}}i\in \left(I^{\mathrm{BECCS}}\cup I^{\mathrm{NS}}\right),r\in R,\forall t\in T$$

Equation 29 ensures the balance between biomethane (*CH*_4_) supply and its utilization as an input to downstream conversion technologies within the modeled energy system. The left-hand side represents the total supply of biomethane available to the market, including direct production from *CH*_4_-producing technologies and the portion of other fuel-producing technologies that contribute to net fuel output (e.g., Bioethanol with CCS). *β*_*i*_ is the fraction (or coefficient) converting technology output *CH*_4_ to net fuel supply (*β*_*i*=*Bioethanol*+*CCS*_ = 0.39). The right-hand side aggregates all technologies that consume biomethane as an input —such as Gas-and-steam turbine power plant, or chemical synthesis pathways such as Ammonia —adjusted by their conversion efficiencies *η*_*t*,*i*_ and $\eta_{t,i}^{\mathrm{chem}}$.

(Equation 29) $$\underset{s\in S_{\mathrm{CH4}}}{\sum }\pi_{t,i,s}+\underset{i,s}{\sum }\left(\beta_{i}\cdot \pi_{t,i,s}\right)=\underset{s,i\in I_{CH4\text{\_}in}}{\sum }\frac{\pi_{t,i,s}}{\eta_{t,i}}+\underset{i\in I_{CH4\text{\_}inChem}}{\sum }\frac{{\pi^{\mathrm{chem}}}_{t,i,s}}{\eta_{t,i}^{\mathrm{chem}}}$$

### Quantification and statistical analysis

Since this is a deterministic optimization research, no statistical tests were performed. Sensitivity analyses conducted (e.g., over carbon price, carbon credit price, storage availability), and the software used (MATLAB, GAMS, CPLEX).
