# Supplementary material for: Unleashing the combined potential of bio-based carbon dioxide removal in Germany’s path to net zero from the bioenergy system perspective
Source: iScience. 2026 Aug 6;29(8):116852. doi: 10.1016/j.isci.2026.116852 (PMC13474250; doi:10.1016/j.isci.2026.116852)
Supplement: Supplementary file 1 [file mmc1.pdf]

iScience, Volume *29*

Supplemental information

Unleashing the combined potential of bio-based carbon dioxide removal in Germany's path to net zero from the bioenergy system perspective

Mohammad Sadr, Danial Esmaeili Aliabadi, Matthias Jordan, Ronja Wollnik, and Daniela Thrän

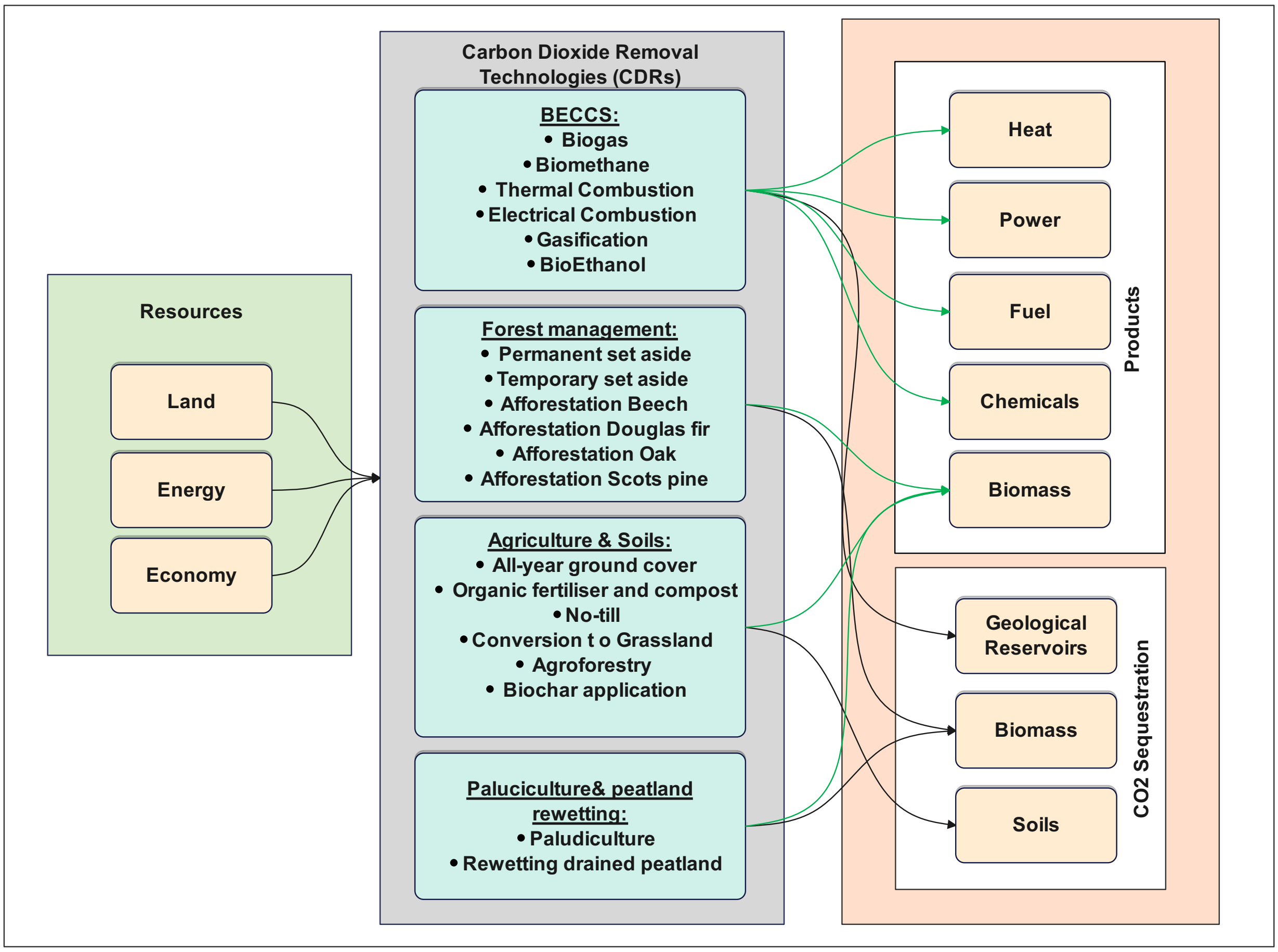

Figure 1: Structure of land-based CDR

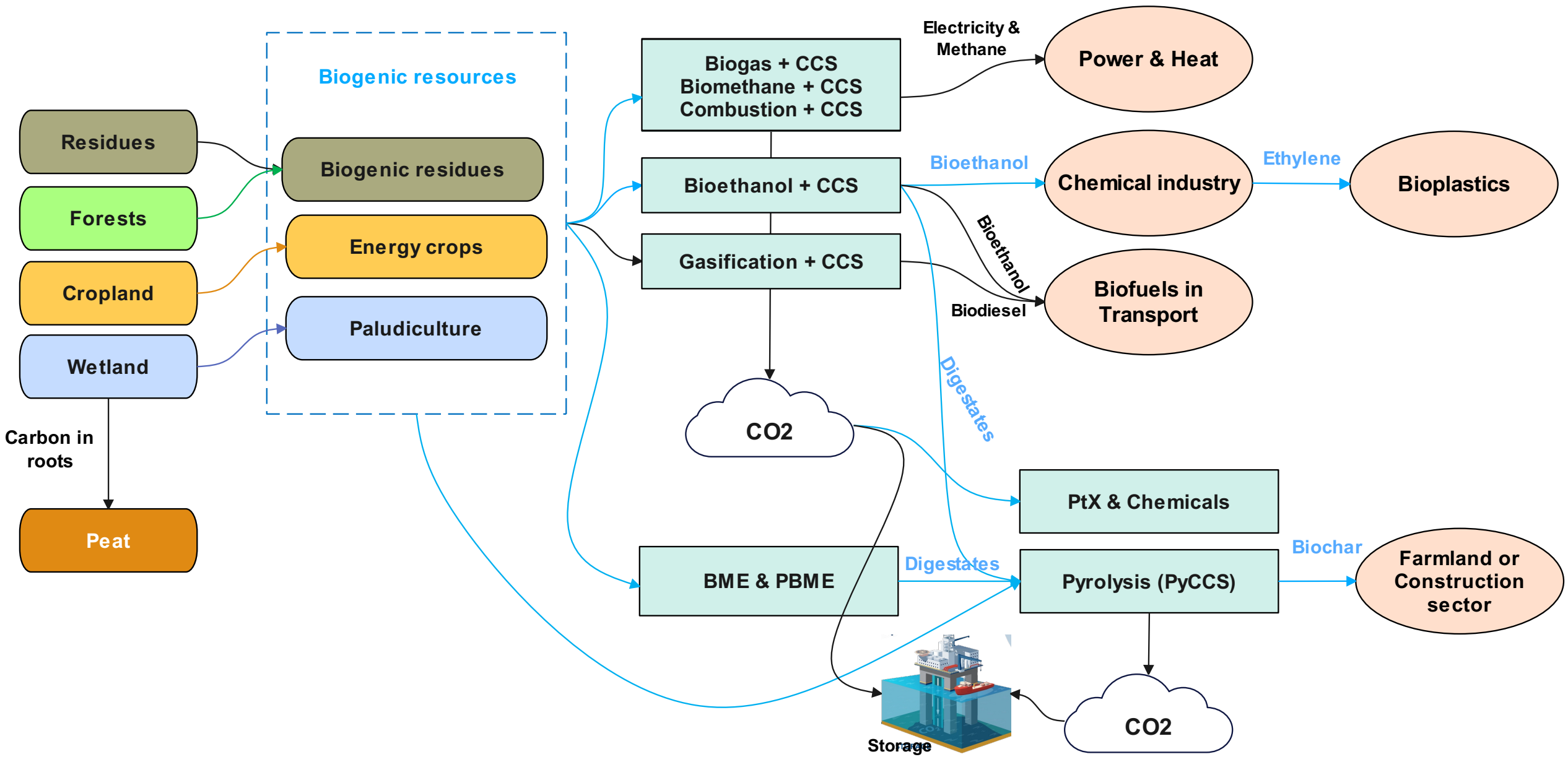

Figure 2: Cascade use of biomass in energy and non-energy sectors, contributing to CDR and CCU (Carbon Capture and Utilization). BME: biomethane based on anaerobic digestion, PBME: power-to-methane via biological methanation, PyCCS: Pyrogenic carbon capture and storage (pyrolysis of waste, straw and residues), CCS: Carbon capture and storage.

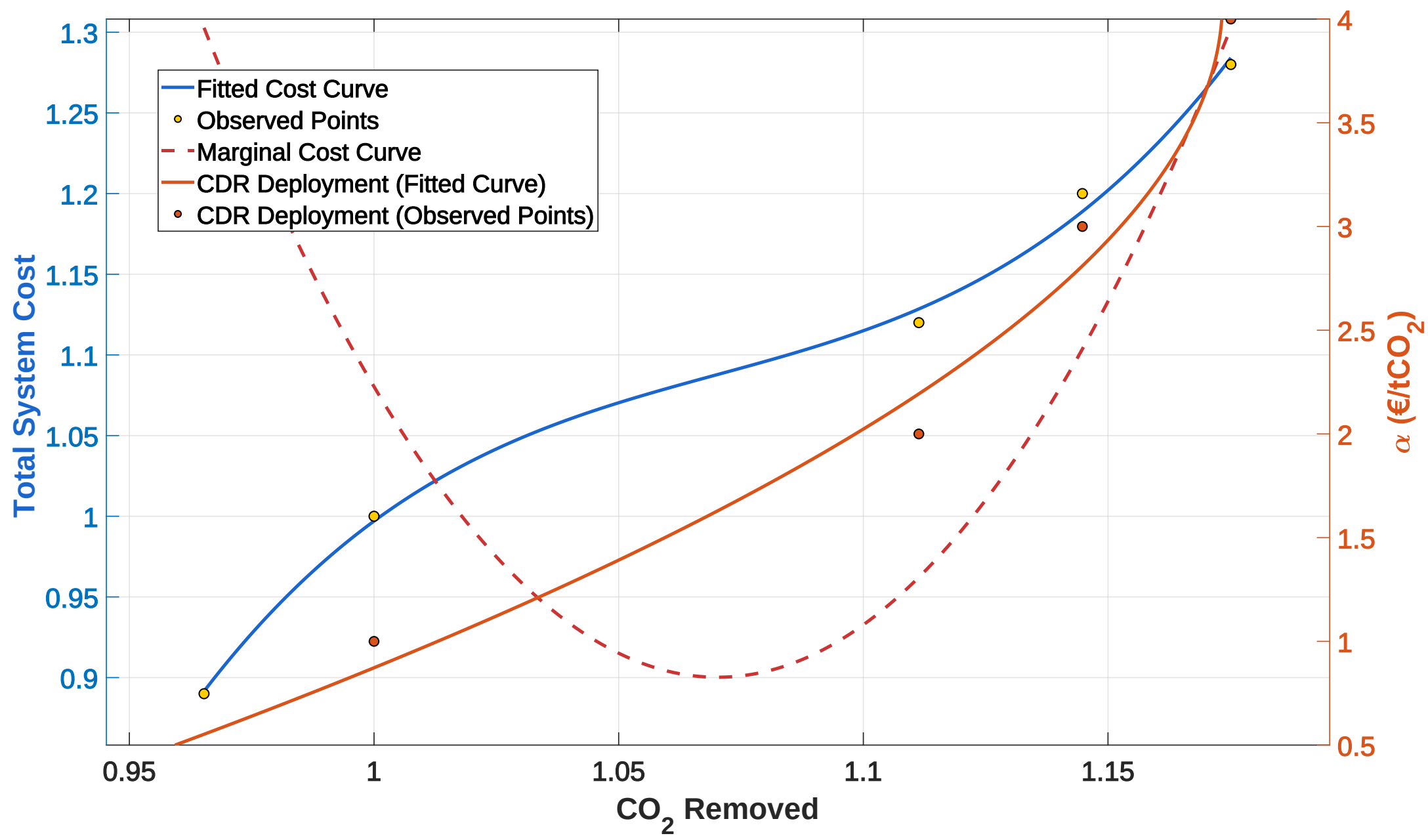

Figure 3: Marginal Removal Cost Curve and system response to carbon price variation.

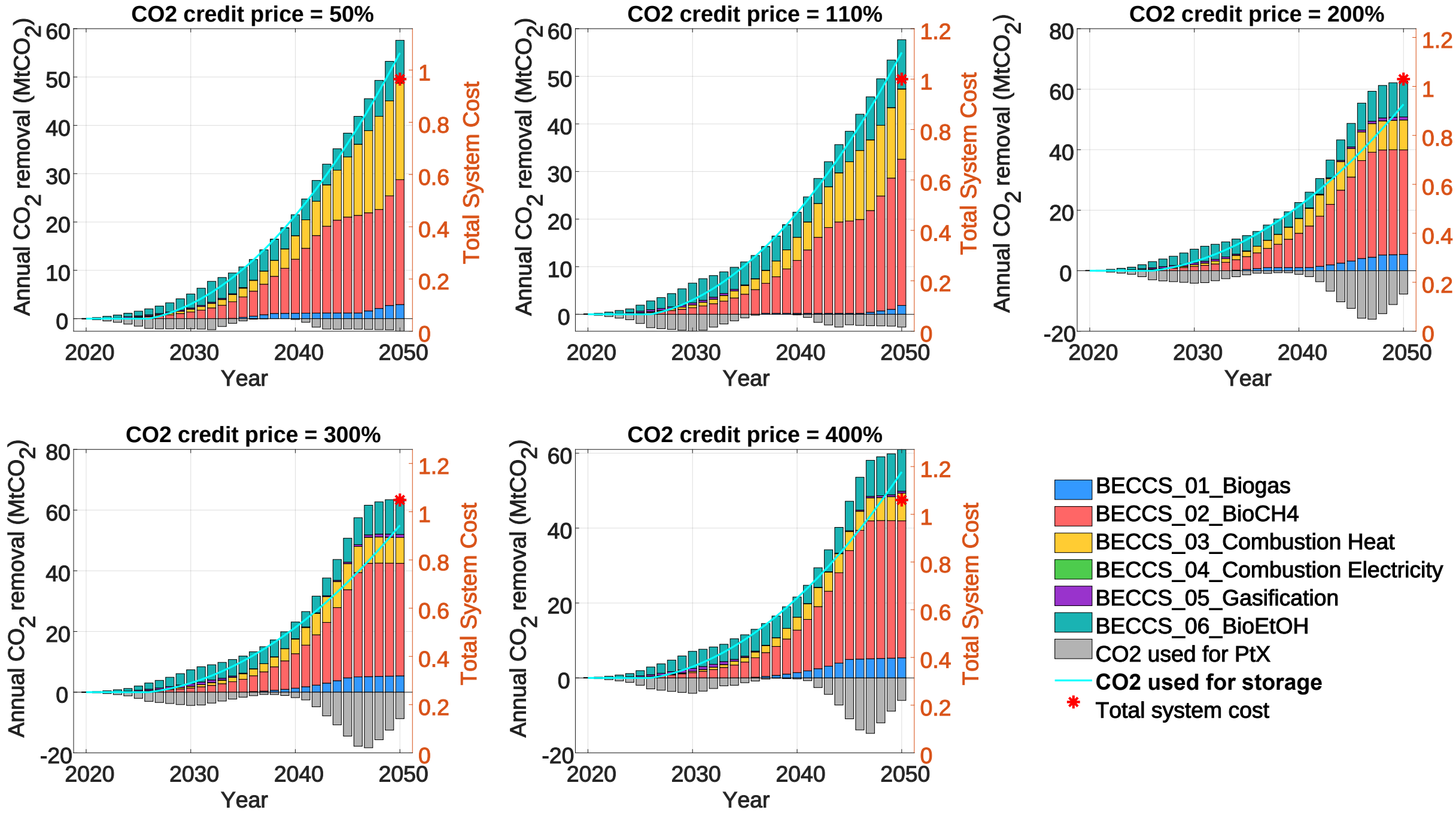

Figure 4: BECCS sensitivity analysis over carbon credit

.

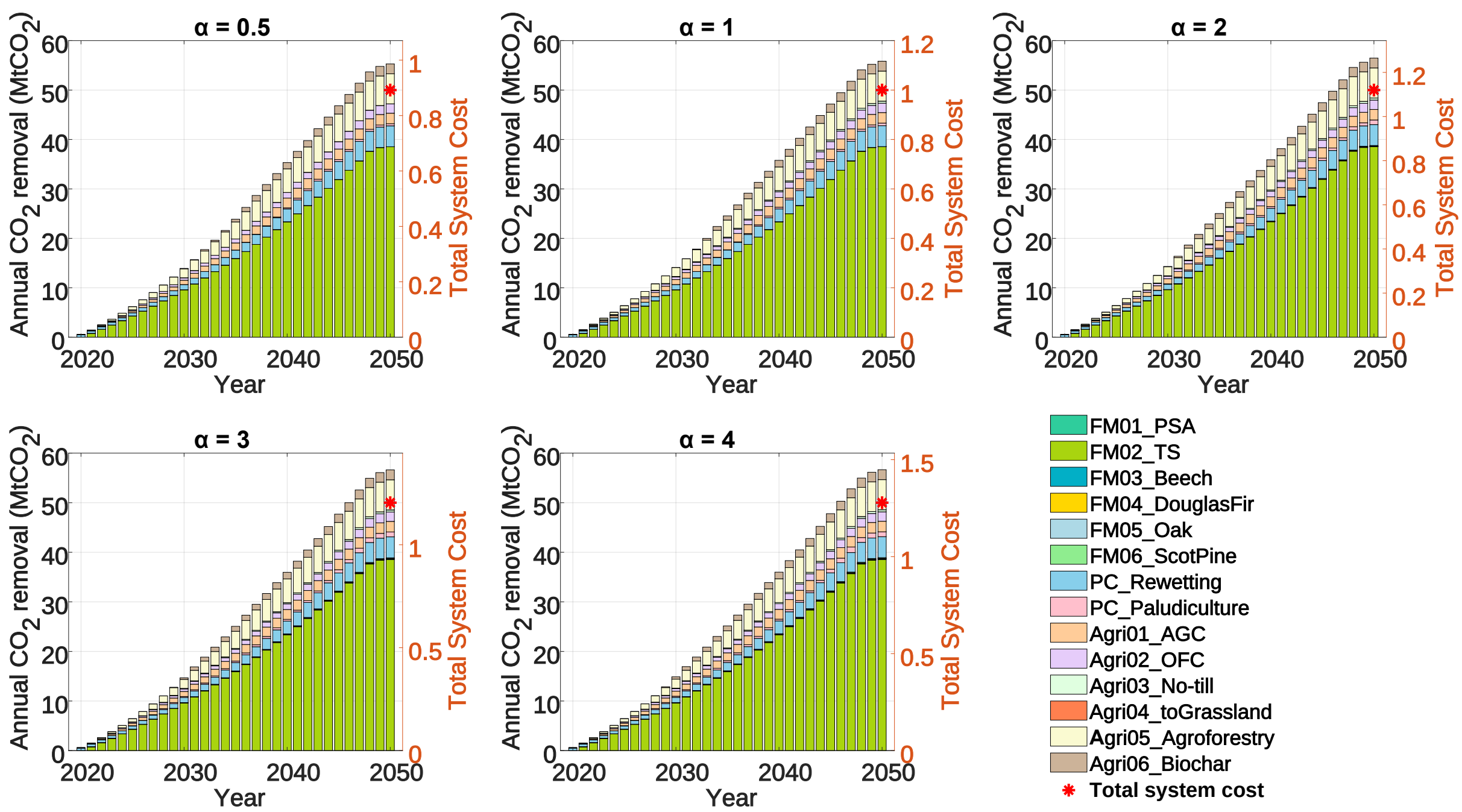

Figure 5: Natural sinks sensitivity analysis over carbon price. The α factor is a multiplier that adjusts the base carbon price ($\alpha = 1$).

Techno-economic parameters

The following tables describe major inputs for the modeling forest-based, wetland (see Table 3) and agriculture-based (see Table 4) concepts in BENOPTex.

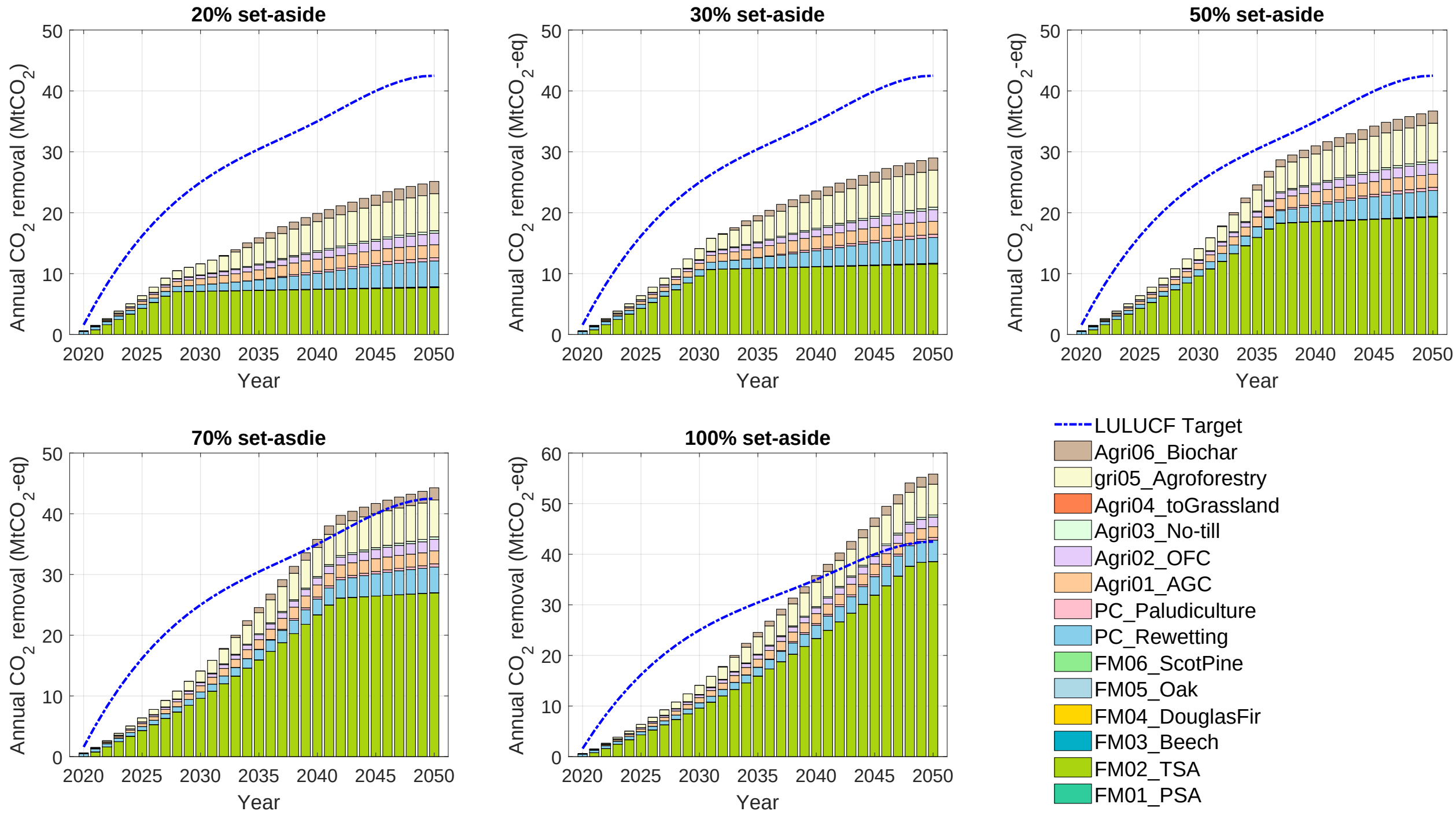

Figure 6: Sensitivity analysis of beech forest set-aside shares on annual CO_2 removal (2020–2050).

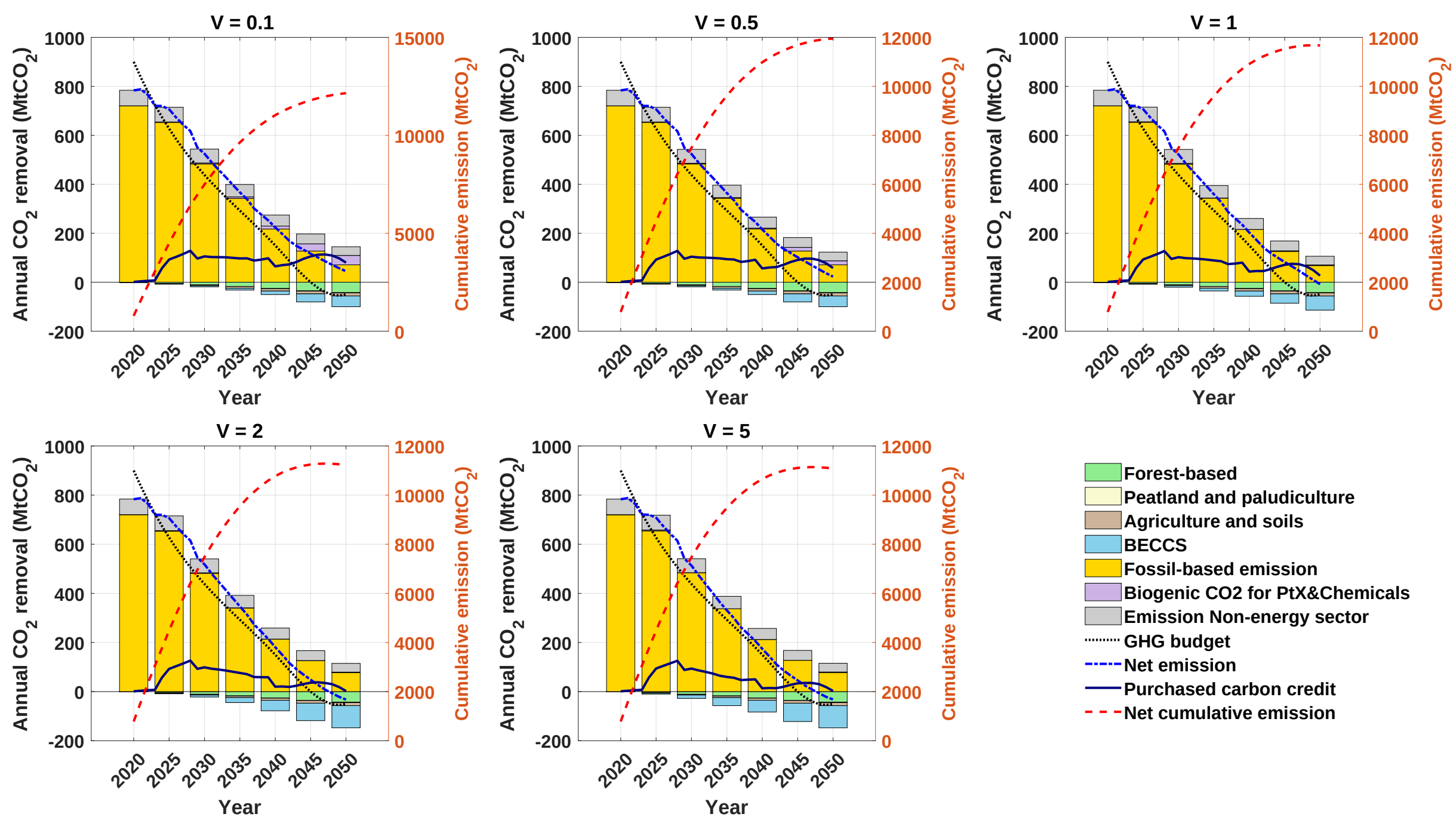

Figure 7: Storage availability system-wide impacts on emissions and CDR deployment. In the base case ($V = 1$), the available storage is projected to reach 55 Mt CO_2 per year by 2050.

In contrast to Sadr et al.[1], we have updated the data related to the biomethane pathway with CCS, as shown in Table 5. These updates were incorporated into the model, and all relevant

Table 1: Summary of optimization results related to natural sinks deployment in Germany

Varriables	Unit	Forest-based and peatland							
		Permanent	Temporary	Beech	DouglasFir	Oak	ScotsPine	Rewetting	Paludiculture
**Removal size in 2050**	$MtCO_2$	–	38,5	–	0.014	–	0.0102	4.23	0.53
**Land size in 2050**	M ha	–	2.39	–	0.0013	–	0.0014	0.69	0.17
**Total cost of implementation**	B Euro	–	27	–	0.053	–	0.012	5.45	–
**Cumulative removal by 2050**	$MtCO_2$	–	544	–	0.19	–	0.024	49	6.13
**aLCOR**	€/ tCO_2	–	50	–	260	–	486	98	–

Table 2: Summary of optimization results related to natural sinks deployment in Germany

Varriables	Unit	Agricultural practices					
		All-year ground cover	Organic fertiliser and compost	No-till	to Grassland	Agroforestry	Biochar
**Removal size in 2050**	$MtCO_2$	2.13	1.9	0.42	–	6	2
**Land size in 2050**	M ha	1.77	2.37	0.2	–	0.44	2.3
**Total cost of implementation**	B Euro	2.03	1.74	1.225	–	2.55	8.05
**Cumulative removal by 2050**	$MtCO_2$	42.5	26.5	5.86	–	99.5	19.40
**aLCOR**	€/ tCO_2eq	64	84	188	–	24	406
**Cumulative natural sinks removal by 2050**	Gt CO_2	0.766					

scenarios were analyzed. The biomethane produced can be utilized for heat or electricity production.

Table 3: Techno-economic parameters of the forest-based and peatland rewetting & paludiculture options

Parameters	Unit	Set-aside concepts		Afforestation concepts				Peatland rewetting and Paludiculture	
		Permanent	Temporary	Beech	DouglasFir	Oak	ScotsPine	Rewetting	Paludiculture
**Max growth rate 2020**	ha/year	55,050	55,050	783	71	360	785	2,000	
**Max growth rate 2050**	ha/year	119,674	119,674	783	71	360	785	50,000	
**Relative CO_2 removal potential**	tCO_2eq/ha/year	14.2	14.2	3.8	9.6	1.1	4.8	6.1	
**Investment $cost_{2020}$**	€/ha	14×10^3	14×10^3	5,832	1,654	4,905	3,950	5,500	
**Investment $cost_{2050}$**	€/ha	36×10^3	16×10^3	7,480	2,122	6,291	5,066	13,825	
**O&M $cost_{2020}$**	% of Investment cost	0	0	1.23	2.7	1.38	0.63	17	
**O&M $cost_{2050}$**	% of Investment cost	0	0	1.58	3.4	1.77	0.81	7	
**Germany's forest types area**	ha	–	–	2,393,484	597,437	1,343,657	2,115,362		
**Germany's grassland area**	ha	–	–	5,105,719					

Table 4: Techno-economic parameters of the agriculture-based options

Parameters	Unit	All-year ground cover	Organic fertiliser and compost	No-till	to Grassland	Agroforestry	Biochar
**Max growth rate 2020**	ha/year	78,500	55,705	4,695	530	5,760	33,367
**Max growth rate 2050**	ha/year	78,500	55,705	4,695	530	35,067	667,333
**Relative CO_2 removal potential**	tCO_2eq/ha/year	1.2	0.8	2.1	3.1	13.8	0.87
**Investment $cost_{2020}$**	€/ha	2,476	2,476	1,111	3,967	2,389	2,476
**Investment $cost_{2050}$**	€/ha	3,929	3,929	1,763	7,506	3,790	3,929
**O&M $cost_{2020}$**	% of Investment cost	10.9	7.5	22.4	9.0	7.7	24.1
**O&M $cost_{2050}$**	% of Investment cost	17.2	11.9	35.6	14.4	12.3	38.2

Table 5: Techno-economic parameters for the BECCS energy plants

Parameters	Unit	BECCS plant types					
		**Biogas**	**Biomethane**	**Combustion_Heat**	**Combustion_Electricity**	**Gasification + Fischer tropsch**	**Bioethanol**
**Rated capacity**	KW	500	2500	800	500,000	100,000	164,000
**Operating hours**	h	8000	8410	5000	7446	7000	7500
**Life time**	a	25	25	25	25	25	25
**Efficiency in 2020**	%	22	53	80	33	39	53
**Efficiency in 2050**	%	22	55	80	34	44	58
**CO_2 separation rate$_{2020}$**	%	90	95	90	90	90	81
**Relative GHG removal potential**	tCO_2eq/GJ	-0.222	-0.190	-0.081	-0.223	-0.048	-0.105
**Investment cost$_{2020}$**	€/KWcap	2735	2040	4843	4300	3000	1390
**O&M cost$_{2020}$**	% of Investment cost	6.51	3.30	5.20	10.30	2.50	6.60
**Capex of CCS unit $_{2020}$**	€/KWcap	8214	2638	1502	7800	771	2201
**O&M cost of CCS unit$_{2020}$**	% of Investment cost	20.36	5.44	2.63	12.1	1.05	8.18
**Output**	Type	Electricity	Biomethane	Heat	Electricity	Diesel & Heat	Ethanol & Biomethane

References

1. Sadr, M., Aliabadi, D.E., Jordan, M., and Thrän, D. (2024). A bottom–up regional potential assessment of bioenergy with carbon capture and storage in Germany. Environmental Research Letters 19, 114047.
